# Ovalbumin-Induced Allergic Inflammation Diminishes Cross-Linked Collagen Structures in an Experimental Rabbit Model of Corneal Cross-Linking

**DOI:** 10.3389/fmed.2022.762730

**Published:** 2022-05-26

**Authors:** Zhongyang Zhao, Minghui Liang, Huan He, Xuemei Wang, Chengfang Zhu, Lan Li, Bin Liu, Rongrong Zong, Qifang Jin, Huping Wu, Wei Li, Zhirong Lin

**Affiliations:** ^1^Eye Institute and Affiliated Xiamen Eye Center of Xiamen University, School of Medicine, Xiamen University, Xiamen, China; ^2^Fujian Provincial Key Laboratory of Ophthalmology and Visual Science, Xiamen, China; ^3^The Second Affiliated Hospital of Nanchang University, Nanchang, China; ^4^Fujian Key Laboratory of Ocular Surface and Corneal Disease, Affiliated Xiamen Eye Center of Xiamen University, Xiamen, China

**Keywords:** allergic conjunctivitis, corneal collagen cross-linking, inflammatory cytokine, matrix metalloproteinase, *in vivo* confocal microscopy (IVCM)

## Abstract

**Background:**

Allergic conjunctivitis (AC) is one of the reported potential risk factors of progression in keratoconus patients after corneal cross-linking surgery; however, the causal relationship is still inconclusive. Recent studies have indicated that various inflammatory cytokines play a vital role in the development of primary keratoconus. It is still unclear whether these inflammatory mediators also trigger CXL failures. This study aimed to investigate the impact of AC on the rabbit corneas after *trans-*epithelial corneal cross-linking (TCXL).

**Methods:**

A total of six rabbits were kept untreated as the normal control (NC) group. A total of 18 rabbits were treated by TCXL and divided into three groups (six in each group), namely, no treatment (TCXL group); induction of AC (TCXL + AC group); and induction of AC plus topical prednisolone acetate (TCXL + AC + PA group), according to additional treatment. AC was induced by topical application of ovalbumin after intraperitoneal pre-sensitization with ovalbumin. Rabbits were evaluated by slit lamp, *in vivo* laser scanning confocal microscopy, anterior segment optical coherence tomography, and measurement of corneal biomechanics. The cornea specimens were collected for the transmission electron microscope, the collagenase I digestion test, and PCR assay for TNF-α, IL-6, IL-1β, matrix metalloproteinase 9 (MMP-9), lysyl oxidase (LOX), and tissue inhibitor of metalloproteinases 1 (TIMP-1) on the day (D) 28.

**Results:**

On D28, the TNF-α, IL-6, IL-1β, MMP-9, and LOX levels were significantly increased while the TIMP-1 was decreased in the TCXL + AC group when compared with the TCXL and TCXL + AC + PA groups. *In vivo* confocal microscopy revealed that at a depth of 150–210 μm, a trabecular patterned hyperdense structure surrounded by elongated needle-like processes could be observed in the TCXL and TCXL + AC + PA groups, but hardly seen in the TCXL + AC group. The demarcation lines were indistinct and blurred in the TCXL + AC group. An electron microscope demonstrated less interlacing fibril lamellae and higher interfibrillar spacing in the TCXL + AC group. The stability of corneal biomechanics and resistance to collagenase were decreased in the TCXL + AC group.

**Conclusion:**

The corneal microstructures induced by TCXL and biomechanical stability were diminished in rabbits with AC but could be maintained by topical anti-inflammatory treatment. Our results supported the causal relationship between altered cytokine profiles and corneal microstructure after primary corneal cross-linking.

## Introduction

In recent years, corneal collagen cross-linking (CXL) has been recognized as a safe and effective treatment to delay or halt the further progression of keratoconus. During CXL, riboflavin interacts as a photosensitizer with ultraviolet-A light to create cross-linking of protein fibrils followed by the formation of interchain disulfide bonds, thus arresting the progression of corneal ectasia by increasing the biomechanical stability of the cornea. Various protocols (1) of CXL have been performed and extensively investigated, showing the long-term efficacy of stabilization and improvement for keratoconus. Conventional CXL with the removal of epithelium has become one of the standard treatments of progressive keratoconus worldwide.

Accumulating evidence suggests a pivotal role for inflammation in the pathogenesis of keratoconus (2). Several studies have shown overexpression of inflammatory cytokines in the tear fluid of patients with keratoconus (3), and the levels of inflammatory factors were associated with corneal topography and even the refractive parameters (4). Altered corneal epithelial and stromal expressions of specific genes, such as tumor necrosis factor-α (TNF-α), interleukin-1 (IL-1), IL-6, and matrix metalloproteinase 9 (MMP-9), were also found at the corneal cone apex in keratoconus (5). Eye rubbing and allergic conjunctivitis are known risk factors for keratoconus (6–8), which could induce increased inflammatory factors, oxidative stress, and matrix metalloproteinase, leading to the degradation of the corneal extracellular matrix and triggering the thinning of the corneal stroma and the loss of biomechanical strength. However, inflammation is just insufficient to guarantee the occurrence of keratoconus, as many patients with severe corneal inflammation do not develop keratoconus. Nevertheless, increasing evidence support the contribution of several inflammatory cytokines or mediators that, in part, orchestrate or amplify corneal tissue damage.

Unfortunately, the risk factors associated with keratoconus progression after a primary CXL have remained unclear in the past decades (9, 10). Theoretically, the risk factors are likely to be similar to those in primary keratoconus, such as eye rubbing. In some studies (11), history of allergic conjunctivitis was considered as a risk factor for CXL failure. However, most of these observations are cross-sectional, which clouds the determination of a causal relationship between altered cytokine profiles and keratoconus progress after a primary CXL. Here, we hypothesized that the allergic inflammatory environment might impair the maintenance of CXL effects. In this study, CXL was performed in rabbits with allergic conjunctivitis induced by ovalbumin, and the structural alterations were evaluated in this study to investigate the role of allergic inflammation after CXL.

## Materials and Methods

All procedures were performed in accordance with the National Institutes of Health guide for the care and use of laboratory animals, and ARVO Statement for the Use of Animals in Ophthalmic and Vision Research. The protocols in this study were approved by the Animal Ethics Committee of Xiamen University.

### Animals and Experimental Procedures

A total of 24 healthy female New Zealand white rabbits (body weight 2.5–3.5 kg, purchased from Shanghai Laboratory Animal Center, Shanghai, China) without clinically observable ocular surface disease were used for this study and held in a standard environment throughout the study as follows: room temperature, 25°C ± 1°C; relative humidity, 65% ± 10%; and alternating 12-h light/dark cycles (7 AM to 7 PM).

### Surgical Procedure for *Trans-*Epithelial Corneal Collagen Cross-Linking

Of these 24 rabbits, six were kept untreated as the normal control (NC) group, while the other 18 rabbits received *trans-*epithelial corneal collagen cross-linking (TCXL). Topical 0.5% proparacaine hydrochloride eye drops were instilled three times before surgery (every 5 min). All surgical procedures (12, 13) were performed in a sterile operating room. The riboflavin solution (ParaCel, Avedro, United States), which was composed of 0.25% riboflavin with hydroxypropyl methylcellulose, ethylenediaminetetraacetic acid (EDTA), and benzalkonium chloride (BAC), was applied to the cornea of the right eye every 1 min for 6 min. Chemicals, such as BAC and EDTA, were utilized to increase the epithelial permeability to riboflavin (14). After the residual riboflavin was rinsed away with normal saline, 0.25% of riboflavin solution without benzalkonium chloride (ViberX Xtra, Avedro, United States) was applied to the cornea every 1 min for 4 min. The central cornea was then irradiated (UVX-2000, IROC, Switzerland) with a light spot of 9 mm diameter for 10 min at a 9 mW/cm^2^ UV-A light (5.4 J/cm^2^ surface dose). Normal saline was further applied to the cornea every 1 min during the UV-A irradiation. At the end of the surgery, ofloxacin eye ointment was applied to the conjunctival sac for once. After TCXL, all the rabbits received ofloxacin eye drops twice daily for 28 days.

### Induction of Allergic Conjunctivitis

Of 18 rabbits, six were treated by TCXL and served as the TCXL group, while the other 12 rabbits were sensitized intraperitoneally with ovalbumin (1.8 mg/kg) (OVA, Sigma Aldrich Corp., China) and aluminum hydroxide (90 mg/kg) (Sigma-Aldrich Corp., China) on day (D) 1 and D7 after TCXL surgery for the induction of allergic conjunctivitis. Then, the sensitized rabbits were challenged with 100 μl of OVA eye drops (4 mg/ml in PBS) daily at 12 AM from D14 to D28 (15). From D14 to D28, six of them further received topical 0.1% prednisolone acetate ophthalmic solution (Allergan Pharmaceuticals Ireland, Ireland) twice daily at 8 AM and 4 PM (TCXL + AC + PA group), while the other six rabbits received no additional anti-inflammatory treatment (TCXL + AC group).

After TCXL, all of the rabbits were evaluated once a week under a slit-lamp microscope for a corneal inflammatory index of the cornea and corneal epithelial staining scores. The rabbits received the final evaluation under the slit lamp after 16 PM on D28 and were then sacrificed by air embolization. The corneas were collected for further evaluation as described below.

### Corneal Fluorescein Sodium Staining

Four quadrants of corneal surface staining were examined using cobalt blue light for 90 s after the corneal surface was stained by the fluorescent paper. The four quadrants were scored as previously described (16): absent, score 0; slight punctate staining with fewer than 30 spots, score 1; punctate staining with more than 30 spots but not diffuse, score 2; severe diffuse staining but no positive plaque, score 3; or positive fluorescein plaque, score 4. The scores of each quadrant were added together to arrive at a final score (16 points total).

### *In vivo* Corneal Confocal Microscopy

Images of the corneal epithelium, stroma, and endothelium were collected using the *in vivo* corneal confocal microscopy (IVCM, HRT3/Rostock Cornea Module, Heidelberg Engineering Inc., Germany) as described previously (17). After the rabbits were anesthetized, a drop of carbomer gel was applied to the lens cap as a coupling medium. The central cornea was maintained in contact with the cap. By controlling the depth of the z-direction at 2 μm increments manually, images from the corneal epithelium to the corneal endothelium were recorded.

### Anterior Segment Optical Coherence Tomography

Anterior segment optical coherence tomography (AS-OCT, Visante OCT, Carl Zeiss Meditec Inc., Germany) was employed to obtain the images of the corneal stroma as described previously (18). In brief, the rabbits were anesthetized and the eyes were mounted in front of the optical scanning probe to obtain the images according to the manufacturer’s manual.

### RNA Isolation and Real-Time PCR

Total RNA of the cornea was extracted using TRIzol reagent (Thermo Fisher Scientific). Reverse transcription was performed with Oligo 18T primers and reverse transcription reagents according to the manufacturer’s protocol (Takara Bio Inc., Shiga, Japan). Quantitative real-time PCR was performed with mRNA special primers as described in Table 1. PCR reactions were performed on a Bio-Rad CFX96 Touch Real-Time PCR Detection System (Hercules, CA, United States) with SYBR Premix Ex Taq (Takara Bio) at 95°C for 10 min, followed by 45 cycles at 95°C for 10 s, 57°C for 30 s, and 75°C for 10 s, after which melt curve analysis was performed at once from 65°C to 95°C. All of the reactions were performed in triplicate, and the average Ct values greater than 38 were considered negative.

**TABLE 1:** Rabbit sequences of primers used for quantitative real-time PCR.

Name	Sense primer	Antisense primer
GAPDH	5′-TGCCACCCACTCCTCTACCTTCG-3′	5′-CCGGTGGTTTGAGGGCTCTTACT-3′
TNF-α	5′-GTCTTCCTCTCTCACGCACC-3′	5′-GCCCGAGAAGCTGATCTGAG-3′
IL-6	5′-CCGGCGGTGAATAATGAGAC-3′	5′-TGAAGTGGATCGTGGTCGTC-3′
IL-1β	5′-GGCAGGTCTTGTCAGTCGTT-3′	5′-CATGGAGAACACCACTTGTTGG-3′
MMP-9	5′-TCGCCGAGATAGGGAACAAG-3′	5′-CGTCTTCACGTCGAACCTCC-3′
TIMP-1	5′-CCGGACAGACGCTAGAGAATC-3′	5′-AAGGTCGGAGTTGCAGAAGG-3′
LOX	5′-GCAACTACATTCTGAAGGTTAGCG-3′	5′-ACTTCAGAACACCAGGCACT-3′

### Transmission Electron Microscopy

Sample preparation for transmission electron microscopy (TEM) was performed according to the method described previously (19). Briefly, the corneas were harvested and fixed immediately in 0.1 M phosphate buffer containing 2.5% glutaraldehyde at 4°C and washed three times in PBS before post-fixing in 1% osmium tetroxide. Subsequently, the samples were dehydrated in 30% and 50% of ethanol and stained with uranyl acetate in 70% ethanol, followed by dehydration with a graded ethanol series. The samples were embedded in resin and cut into ultrathin sections (70 nm) followed by staining with lead citrate. Sections were examined and photographed at 80 kV with a transmission electron microscope (HT-7800, HITACHI, Japan).

### Analysis of Corneal Biomechanics and Resistance to Collagenase

On D28, the properties of corneal biomechanics in rabbits were measured using Ocular Corvis^®^ ST (Oculus Optikgeräte GmbH, Wetzlar, Germany, equipped with Corvis^®^ ST software version V1.3rx). According to the manufacturer’s instructions, corneal biomechanical parameters, including the maximal deformation amplitude ratio (2 mm from the apex) (DA Ratio Max), were taken by the same experienced technician (20). The digestion assay of corneal tissues was performed using collagenase I (Yeasen, China) at a concentration of 650 U/ml at 37°C for 6 h. The corneal weight was measured before and after the digestion assay. The relative weight was obtained by normalizing it to the initial weight.

### Statistical Analysis

Analysis of the significance of differences between groups was performed using one-way ANOVA followed by a *post hoc* analysis using Tukey’s test to compare the differences between the groups. *P* < 0.05 was considered statistically significant.

## Results

### Clinical Observation of Rabbit Study Group

**FIGURE 1:**
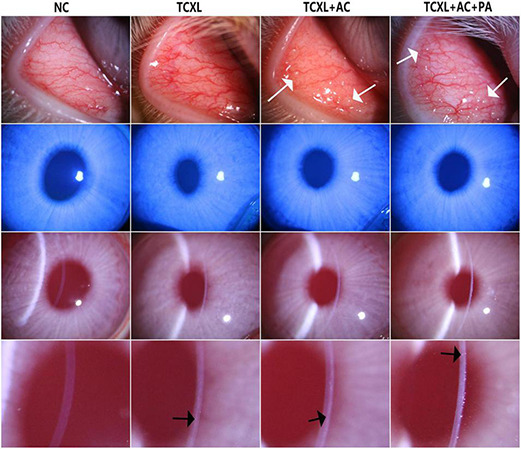
Representative slit-lamp photographs on D28 show the palpebral conjunctival hyperemia (row 1), corneal fluorescein sodium staining (row 2), central corneal edema (row 3) and demarcation line in the anterior stroma (row 4) in different groups. The degree of conjunctival hyperemia in the TCXL + AC group was apparently higher than that in the TCXL group or TCXL + AC + PA group, while no obvious hyperemia was observed in the NC group. No obvious corneal epithelium staining or corneal edema was observed in any group on D28. The white arrows indicated the conjunctival papillae, which were the signs of allergic conjunctivitis. The black arrows indicated the clear demarcation lines in the middle stroma of TCXL and TCXL + AC + PA group, as well as an indistinct hyper-reflective structure in the superficial stroma of TCXL + AC group.

From D1 to D28, no differences in corneal inflammatory index or corneal fluorescein staining scores were found among groups that received TCXL. On D1 after surgery, mild corneal epithelial punctate staining and mild central edema could be observed in all rabbits that received TCXL. However, no obvious corneal epithelium staining or corneal edema was observed in any group from D2 to D28. From D14 to D28, the signs of allergic conjunctivitis were manifested in all the rabbits of the TCXL + AC and TCXL + AC + PA groups. The degree of conjunctival hyperemia in the TCXL + AC group was apparently higher than that in the TCXL group or TCXL + AC + PA group, while no obvious hyperemia was observed in the NC group. The conjunctival papillae as signs of allergic conjunctivitis could be observed in the TCXL + AC and TCXL + AC + PA groups. Clear demarcation lines could be observed in the middle corneal stroma of all the rabbits in the TCXL and TCXL + AC + PA groups, while only indistinct hyper-reflective linear structures in the very superficial stroma of the TCXL + AC group could be seen (Figure 1).

### mRNA Expression of Inflammatory Factors and MMP-9 in Cross-Linked Corneas With or Without Allergic Conjunctivitis

**FIGURE 2:**
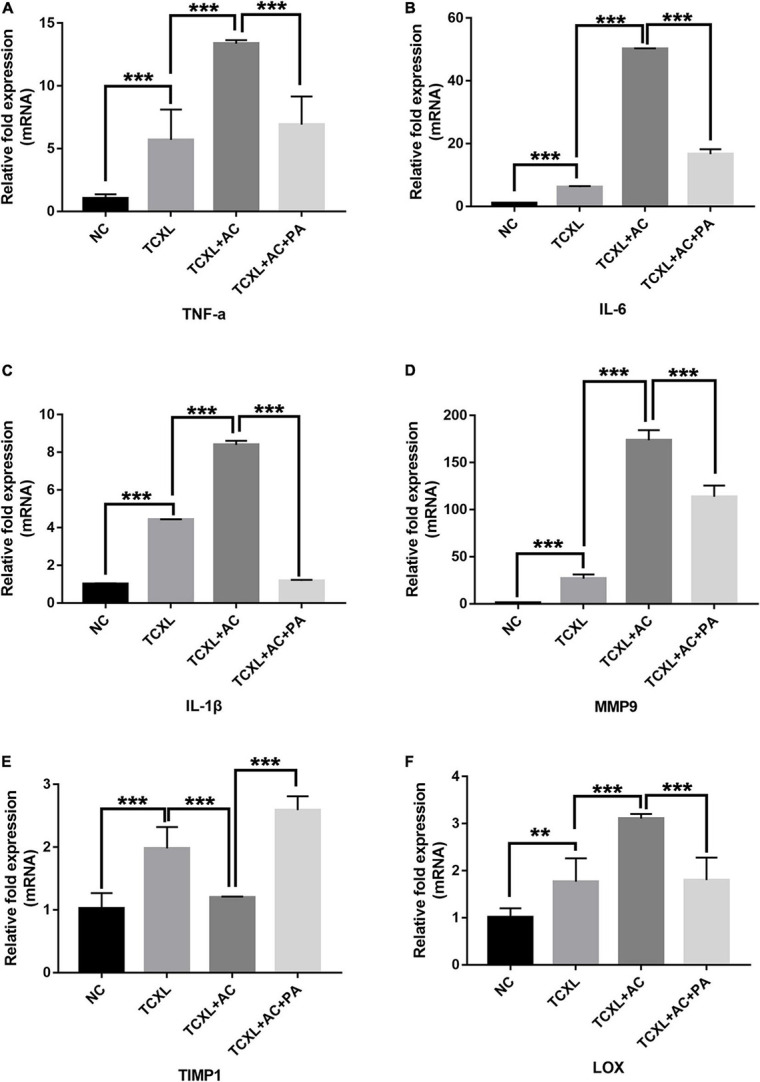
Quantitative analysis of TNF-α **(A)**, IL-6 **(B)**, IL-1β **(C)**, MMP9 **(D)**, LOX **(E)**, and TIMP-1 **(F)** mRNA transcripts in the corneal stroma on D28. The mRNA levels of TNF-α, IL-6, IL-1β, and MMP-9 were significantly increased in the TCXL + AC group when compared with the TCXL and TCXL + AC + PA groups. In contrast, the TIMP-1 level was significantly lower in the TCXL + AC group when compared with the TCXL and TCXL + AC + PA groups. Data are presented as mean ± SD, *n* = 6/group (**P < 0.01; ***P < 0.001).

Quantitative analysis of mRNA transcripts in the corneal stroma on D28 showed that the levels of TNF-α, IL-6, and IL-1β were significantly increased in the TCXL + AC group when compared with the TCXL and TCXL + AC + PA groups (Figures 2A–C). On the contrary, MMP-9 was elevated, while the TIMP-1 levels were decreased in corneas with allergic conjunctivitis (Figures 2D,E). Interestingly, the LOX level was also elevated in corneas with allergic conjunctivitis but reduced by prednisone acetate treatment (Figure 2F).

### Partially Loss of TCXL-Induced Structural Alteration in Rabbit With Allergic Conjunctivitis

**FIGURE 3:**
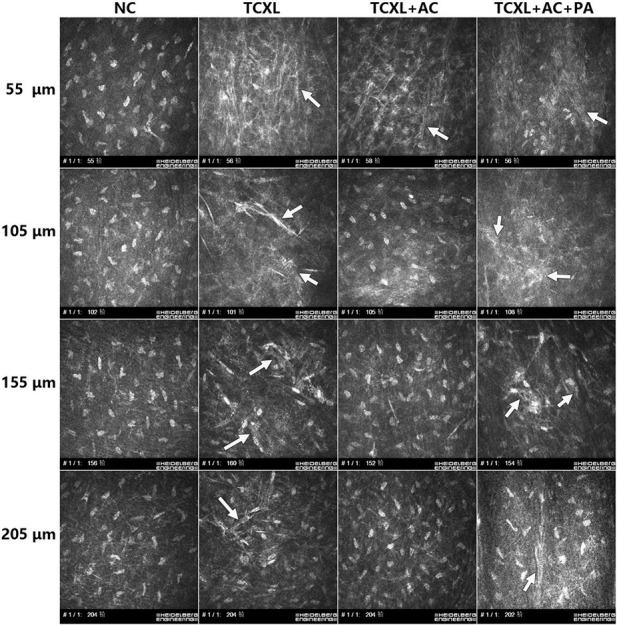
Representative IVCM images showing collagen structures in the anterior corneal stroma on D28 at different depths (row A, 50–60 μm; row B, 100–110 μm; row C, 15–160 μm; and row D, 200–210 μm). At the depths of 150–160 μm and 200–210 μm, trabecular patterned hyperdense structure surrounded by elongated needle-like processes could be observed in the TCXL and TCXL + AC + PA groups (white arrows), but hardly seen in the TCXL + AC group.

On D28, IVCM data revealed the trabecular patterned hyperdense structure with abundant needle-like processes in TCXL, TCXL + AC, and TCXL + AC + PA groups at the depth up to 110 μm. However, these structures could still be observed in the TCXL and TCXL + AC + PA groups, but hardly seen in the TCXL + AC group at depth up to 150–160 μm and 200–210 μm. Representative IVCM images of the morphological alteration of collagen in the anterior corneal stroma on D28 at different depths are shown in Figure 3.

**FIGURE 4:**
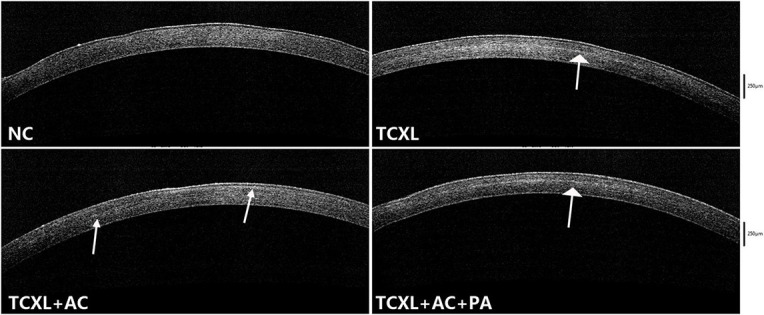
Representative images of anterior segment optical coherence tomography showing the demarcation line (white arrows) in the corneal stroma on D28. No demarcation line could be observed in the normal control. The demarcation lines in the TCXL and TCXL + AC + PA groups were distinct and homogeneous, but indistinct and blurred in the TCXL + AC group.

AS-OCT data revealed that no demarcation line could be observed in the NC group. On D28, the demarcation lines in the TCXL and TCXL + AC + PA groups were distinct and homogeneous but indistinct with weak reflection in the TCXL + AC group (Figure 4).

### Diminishment of Interlacing Corneal Lamellae in Rabbit With Allergic Conjunctivitis

**FIGURE 5:**
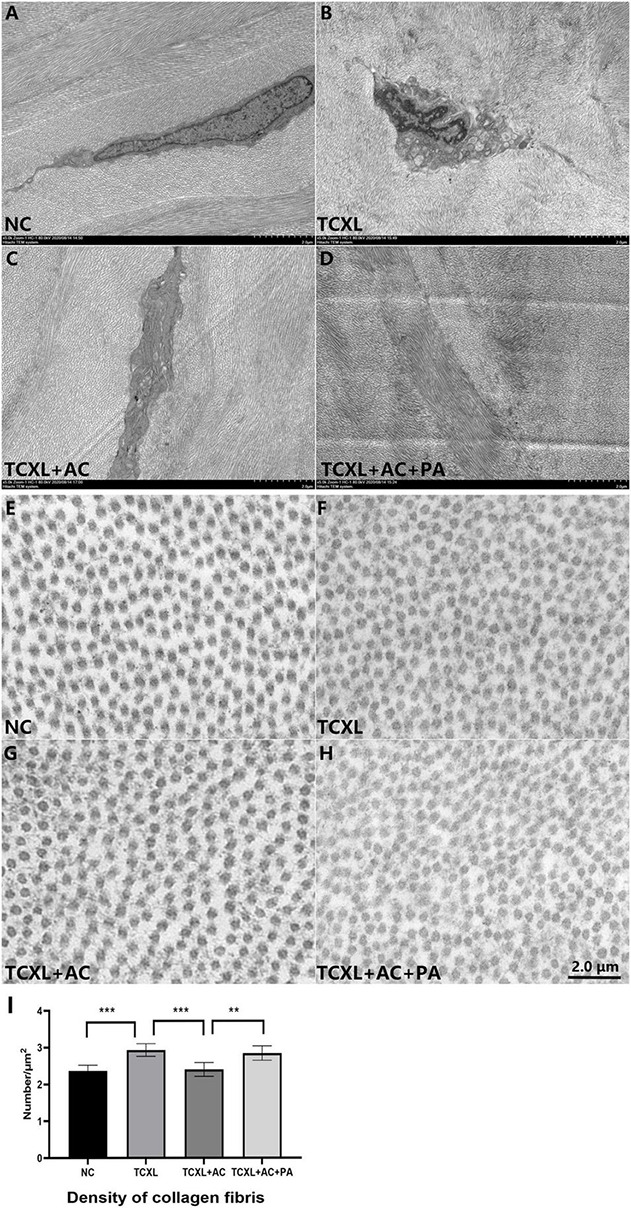
Representative images of transmission electron microscopy showing the ultrastructural changes in corneal stroma on D28. Parallel running collagen fibril lamellae were present in the anterior stroma of normal control **(A)**, while obviously interlacing lamellae were present in the TCXL group **(B)**. In TCXL + AC group, parallel running and interlacing fibril lamellae were both present **(C)**, but the fibril lamellae were still obviously interlacing in TCXL + AC + PA group **(D)**. Cross section of the collagen fibrils showing the interfibrillar spacing in the TCXL cornea **(F,I)** was lower than that of normal control **(E,I)** and TCXL+AC group **(G,I)**, but was comparable with that of TCXL+AC+PA group **(H,I)**. **(A–D)** Magnification 5000×; **(E–H)** magnification 45000×.

Parallel running collagen fibril lamellae were present in the anterior stroma of the normal cornea, while obvious interlacing lamellae were present in the TCXL group. In the TCXL + AC group, parallel running and interlacing fibril lamellae were both present, but the fibril lamellae were still obviously interlaced in the TCXL + AC + PA group. Cross-section of the collagen fibrils shows the interfibrillar spacing in the TCXL cornea was lower than that of normal control and TCXL + AC groups but was comparable with that of the TCXL + AC + PA group (Figure 5).

### Reduction of Corneal Biomechanical Stability in Rabbit With Allergic Conjunctivitis After Corneal Cross-Linking

**FIGURE 6:**
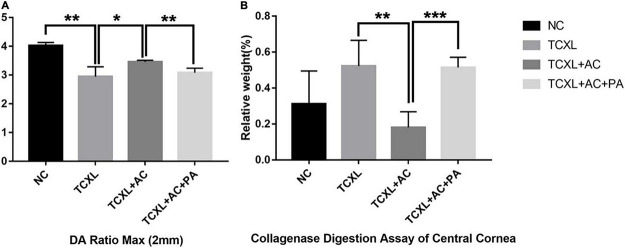
Representative images showing the DA Ratio Max (2 mm) **(A)** and corneal resistance to collagenase digestion **(B)** on D28. The DA Ratio Max of the TCXL group was significantly increased when compared with the NC group, while the DA Ratio Max of the TCXL + AC group significantly decreased when compared with the TCXL group. In the collagenase digestion assay, the final relative weight of the cornea in the TCXL group was significantly higher than those in the NC and TCXL + AC groups. Data were presented as mean ± standard deviation (*n* = 6; *P < 0.05; **P < 0.01; ***P < 0.001).

On D28, DA Ratio Max (2 mm) was (3.46 + 0.05) in the TCXL + AC group, which was significantly higher than in the TCXL group (2.95 + 0.34) (*P* < 0.05) and in the TCXL + AC + PA group (3.08 + 0.15) (*P* < 0.01), still lower than in the NC group (4.03 + 0.11). High DA Ratio Max indicated the decreased corneal rigidity under allergic conditions (Figure 6A).

In digestion assay, the relative weight of cornea in the TCXL group (0.52 ± 0.14), which was almost the same as that in the TCXL + AC + PA group (0.52 ± 0.06), was significantly higher than that in the TCXL + AC group (0.18 ± 0.09) (*P* < 0.01). The relative weight of the normal cornea was 0.32 ± 0.18 (Figure 6B).

## Discussion

The rate of failure or progression after CXL has been reported in many studies, and the mechanism remains unclear. Allergic conjunctivitis and eye rubbing are the two reported potential risk factors for progression in keratoconus patients after CXL. Although recent studies have indicated that various inflammatory cytokines, such as interleukins, play a vital role in the development of primary keratoconus, it is still unclear whether these inflammatory mediators also trigger CXL failures. In this experimental study, the cross-linked corneal collagen structures decreased or diminished after *trans-*epithelial CXL in allergic rabbits with elevated inflammatory cytokines induced by ovalbumin, while the application of anti-inflammatory prednisolone acetate was able to maintain the structural effects induced by CXL. Despite the lack of animal models of keratoconus to date, our results may further support the causal relationship between altered cytokine profiles and corneal microstructure, as well as corneal biomechanical stability after primary corneal cross-linking.

Degradation of corneal stroma in primary keratoconus involves the expression of inflammatory mediators, including proinflammatory cytokines and cell adhesion molecules, which modulate MMP activity and are themselves modulated by it (21). The role of MMPs in keratoconus has been extensively investigated, and any imbalance in the enzymatic activity in the corneal tissue may contribute to the development of keratoconus. The activity of MMPs is regulated by a variety of interleukins and TNF-α (22) and has been implicated in the degradation of type I and IV collagen fibers in ocular disorders. MMPs and cytokines have been found to exhibit complex interactions with each other. IL-1β, IL-6, and TNF-α can stimulate the production of several MMPs. Levels of MMP-9 can inactivate IL-1β (23). The enzymatic activity is a result of the balance with its inhibitors TIMP (24). Many studies have revealed that the cornea after CXL treatment has high stiffness and resistance to enzymatic digestion, which in turn results in lengthening of the turnover time of collagen fibers (25). However, if the enzymatic activity was increased by endogenous or exogenous factors, such as increased inflammatory cytokines, this new-formed balance would be broken once again, leading to the possibility of keratoconus progression after CXL. In our study, elevated IL-1β, IL-6, TNF-α, and MMP-9 mRNA level was found in corneas of the TCXL + AC group accompanied by apparent loss of hyperdense trabecular structure in IVCM, indicating the weakening of CXL effects under the allergic condition of the ocular surface. On the contrary, topical cyclosporine A treatment has been found to be able to reduce MMP-9 levels measured in tears with concomitant arrest of disease progression for primary keratoconus patients (3). Our data revealed that the downregulation of corneal MMP-9, TNF-α, and IL-1β by the anti-inflammatory therapy using prednisolone acetate was accompanied with the attenuation of loss of cross-linked structures. Based on this, long-term postoperative topical anti-inflammatory treatment may have the potential to maintain CXL effect for keratoconus patients who had received CXL. Further basic and clinical investigations are needed to prove this hypothesis since the experimental model used in our study was not a keratoconus animal model.

Eye rubbing, family history of keratoconus, allergy, asthma, and eczema were the most important risk factors for keratoconus according to the available evidence (26). Obviously, most of these risk factors are directly linked to allergic inflammation. The mechanisms through which eye rubbing is followed by corneal remodeling lies in several pathways (27), including the mechanical rubbing microtrauma and the friction between the palpebral conjunctiva and the corneal epithelium, elevated corneal epithelial temperature secondary to friction with the palpebral conjunctiva, the increased distending force on the cornea under elevated intraocular pressure, and the increased release of inflammatory mediators. Eye rubbing was considered as a cause of recurrent keratoconus in some studies (11) and case reports (7). However, no eye rubbing or scratching was observed during the period of allergy induction in our study, while elevated TNF-α, IL-1β, and IL-6 mRNA levels were still confirmed. These results implied that the diminishment of cross-linked fibers was probably associated with the allergic inflammatory responses but not the mechanical rubbing or friction with the palpebral conjunctiva. Allergic conjunctivitis was a major contributing factor to the weakening of CXL structural effect in this experimental model. On the contrary, allergic inflammation may also play in part as an aggravating factor in the pathogenesis of keratoconus. However, chronic inflammation is only insufficient to guarantee the occurrence of keratoconus, as most patients with severe corneal inflammation, such as allergic conjunctivitis, dry eye, and autoimmune ocular surface diseases, do not develop keratoconus. And the population incidence of keratoconus is definitely much lower than those of allergic conjunctivitis and dry eye. However, whether other types of inflammation could also trigger CXL attenuation or even failure need further study.

On the contrary, LOX as a copper-dependent amine oxidase is responsible for the development of lysine-derived cross-links in extracellular matrix proteins (28), such as collagens. Some study has indicated that a reduction in LOX may trigger a reduced production of collagens (29). However, our data showed higher LOX mRNA levels in the TCXL + AC group and lower in the TCXL and TCXL + AC + PA groups. In fact, the role of LOX in KC is proposed by two conflicting reports, suggesting reductions (29) and increases in KC (30). However, it has yet to be determined how LOX activity is altered in corneas after cross-linking and whether the deregulation of LOX contributes to the structural alteration associated with AC.

The depth of the acellular zone of anterior corneal stroma after CXL has been correlated with the effectiveness of the CXL treatment (31, 32). For instance, the limited penetration depth of riboflavin ranged from 100 μm to 240 μm detected in most clinical studies (31) is one of the critical inadequacies of *trans-*epithelial CXL, while the penetration depth was about 300 μm in conventional CXL with epithelial removal. Our IVCM data showed that the penetration depth in the TCXL + AC group was shallower when compared to those in the TCXL and TCXL + AC + PA groups. In fact, the demarcation line revealed by AS-OCT in the TCXL + AC group was indistinct and discontinuous. TEM also showed the loosening between collagen fibrils in the stroma of the TCXL + AC group. The corneal biomechanical strength and resistance to collagenase digestion significantly decreased in the TCXL + AC group, however, could be largely maintained by prednisolone acetate treatment. These results might indicate the further loss of corneal cross-linked structures under the allergic condition on the ocular surface, but longer follow-up time is needed to prove.

## Conclusion

Allergic conjunctivitis could diminish the cross-linked collagen structures by *trans-*epithelial CXL, indicating the weakening of the CXL efficacy. The loss of cross-linked collagen structures and the decrease in corneal biomechanical stability could be partially prevented by topical anti-inflammatory intervention. Further investigation is needed to determine the role of postoperative topical anti-inflammatory treatment in maintaining the CXL effect in keratoconus patients who have received CXL.

## Data Availability Statement

The original contributions presented in the study are included in the article/Supplementary Material, further inquiries can be directed to the corresponding author/s.

## Ethics Statement

The animal study was reviewed and approved by Animal Ethics Committee of Xiamen University.

## Author Contributions

ZL and WL contributed to conceptualization and writing, reviewing, and editing the manuscript. ZZ and ML contributed to methodology, formal analysis, and original draft preparation. HH, XW, CZ, LL, BL, RZ, QJ, and HW investigated and curated the data. All authors have read and agreed to the submitted version of the manuscript.

## Conflict of Interest

The authors declare that the research was conducted in the absence of any commercial or financial relationships that could be construed as a potential conflict of interest.

## Publisher’s Note

All claims expressed in this article are solely those of the authors and do not necessarily represent those of their affiliated organizations, or those of the publisher, the editors and the reviewers. Any product that may be evaluated in this article, or claim that may be made by its manufacturer, is not guaranteed or endorsed by the publisher.
