# Supplementary figures and images for: Ovalbumin-Induced Allergic Inflammation Diminishes Cross-Linked Collagen Structures in an Experimental Rabbit Model of Corneal Cross-Linking
Source: Front Med (Lausanne). 2022 May 26;9:762730. doi: 10.3389/fmed.2022.762730 (PMC9178108; doi:10.3389/fmed.2022.762730)

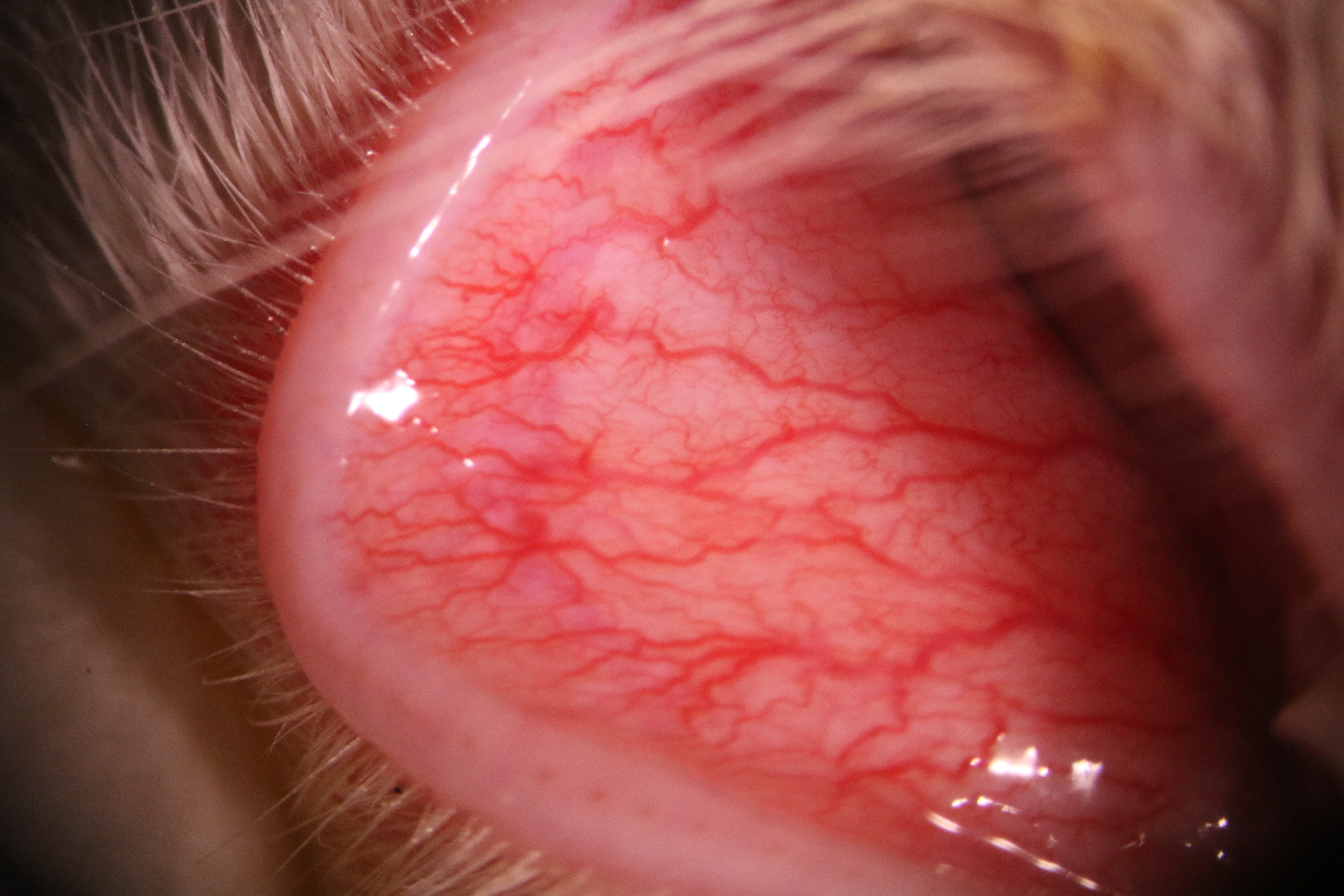

Supplement: Supplementary file 1 [file Data_Sheet_1.ZIP › conjunctival palillary by allergy/TCXL.JPG]

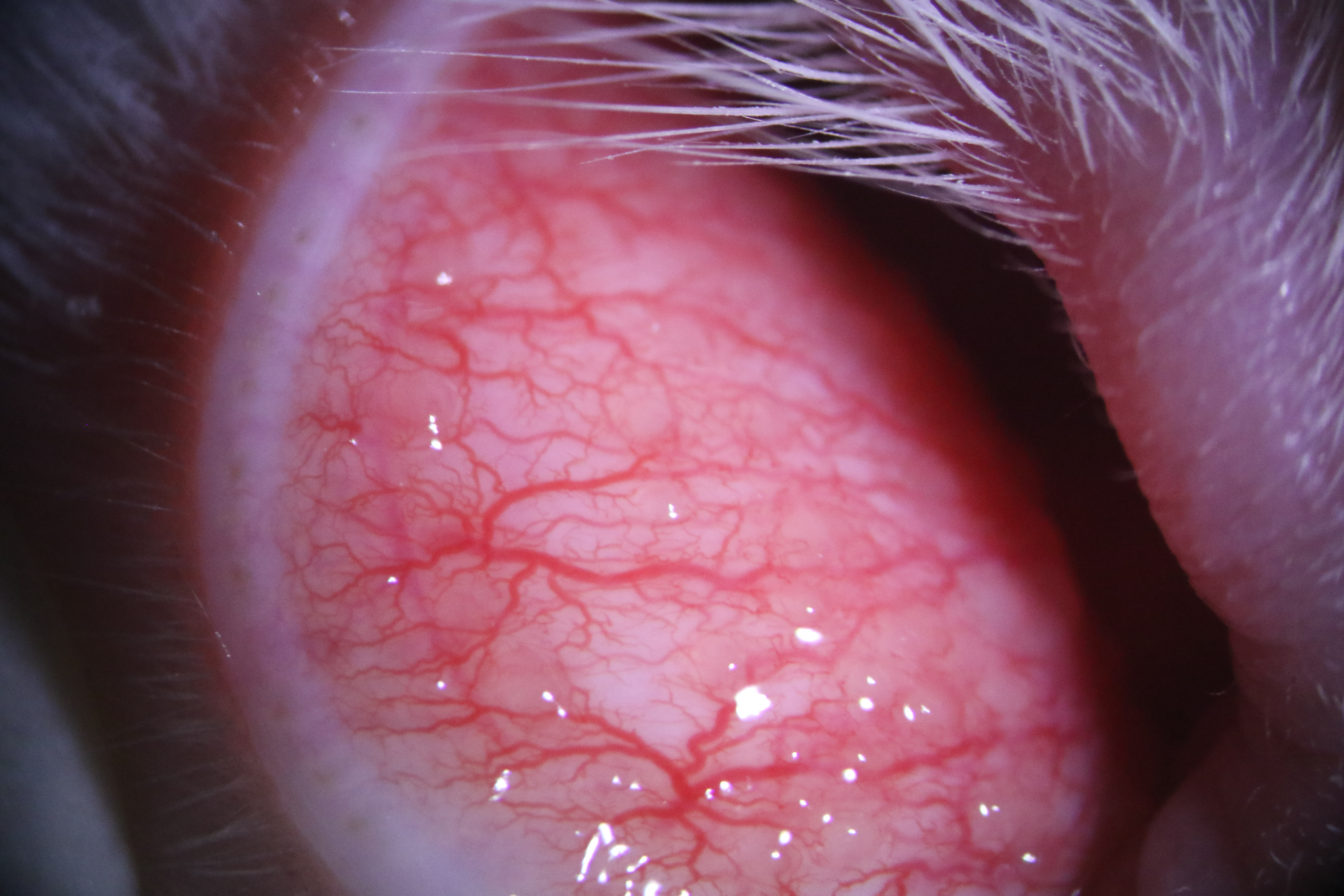

Supplement: Supplementary file 1 [file Data_Sheet_1.ZIP › conjunctival palillary by allergy/TCXL+AC+PA.JPG]

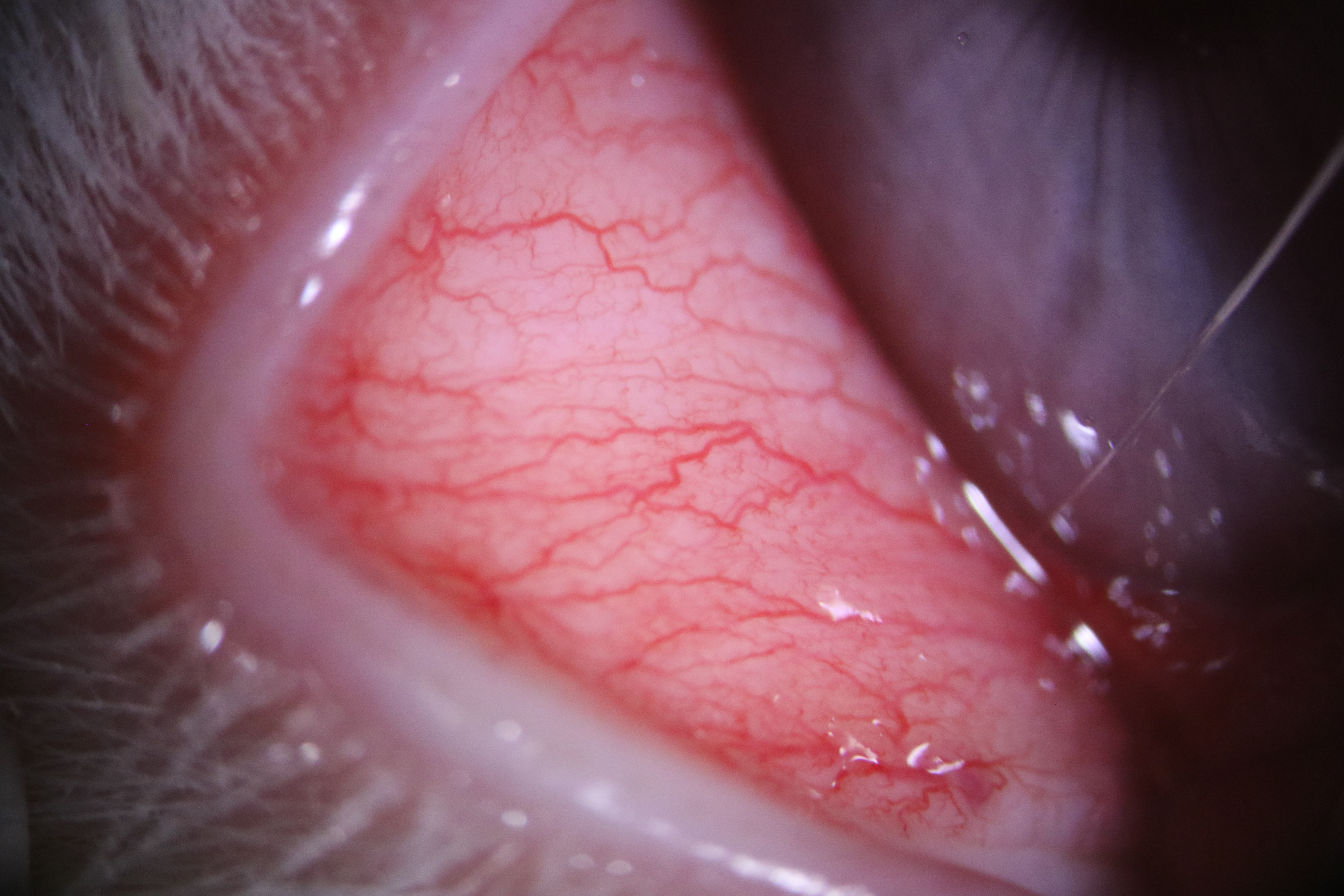

Supplement: Supplementary file 1 [file Data_Sheet_1.ZIP › conjunctival palillary by allergy/NC.JPG]

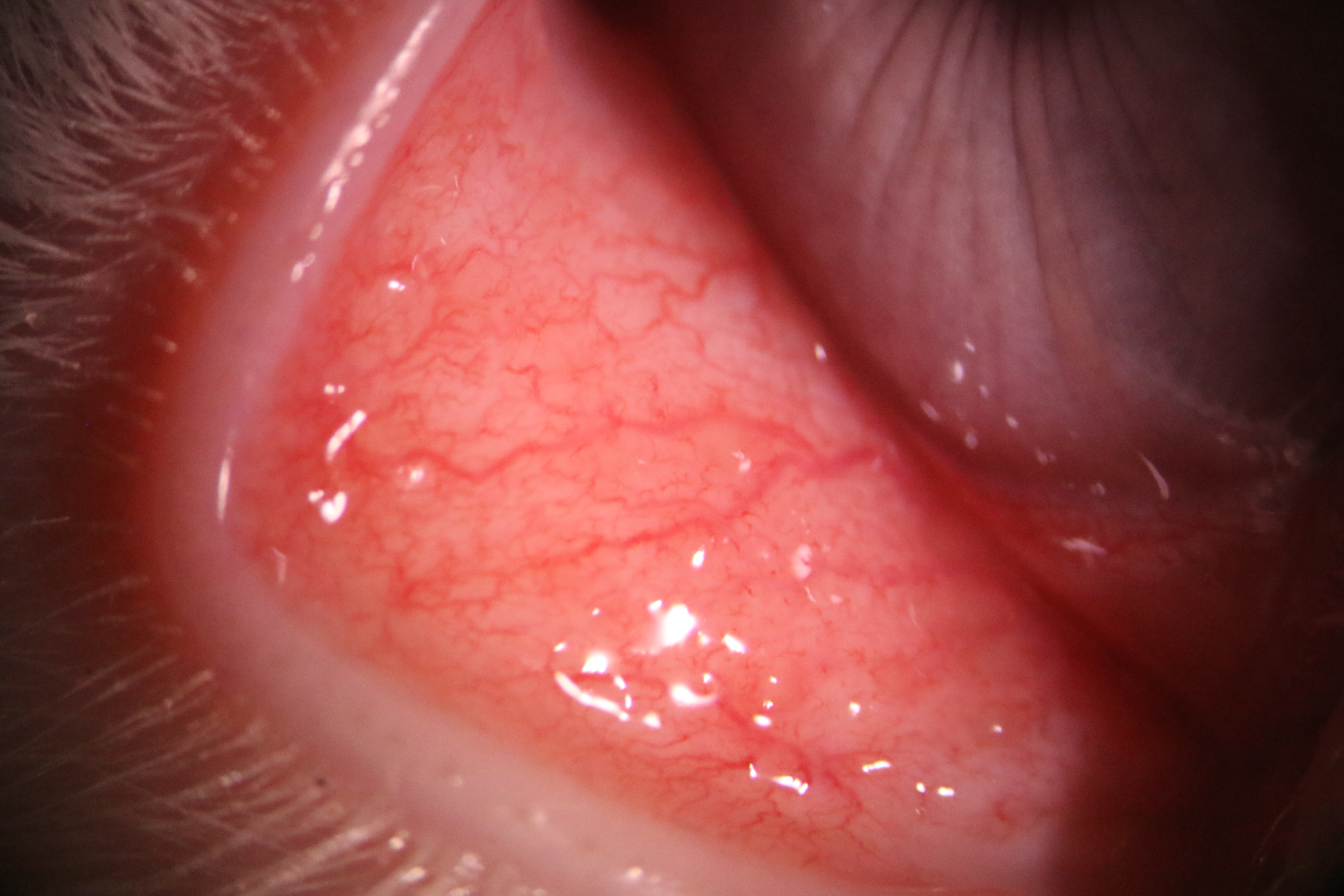

Supplement: Supplementary file 1 [file Data_Sheet_1.ZIP › conjunctival palillary by allergy/TCXL+AC.JPG]

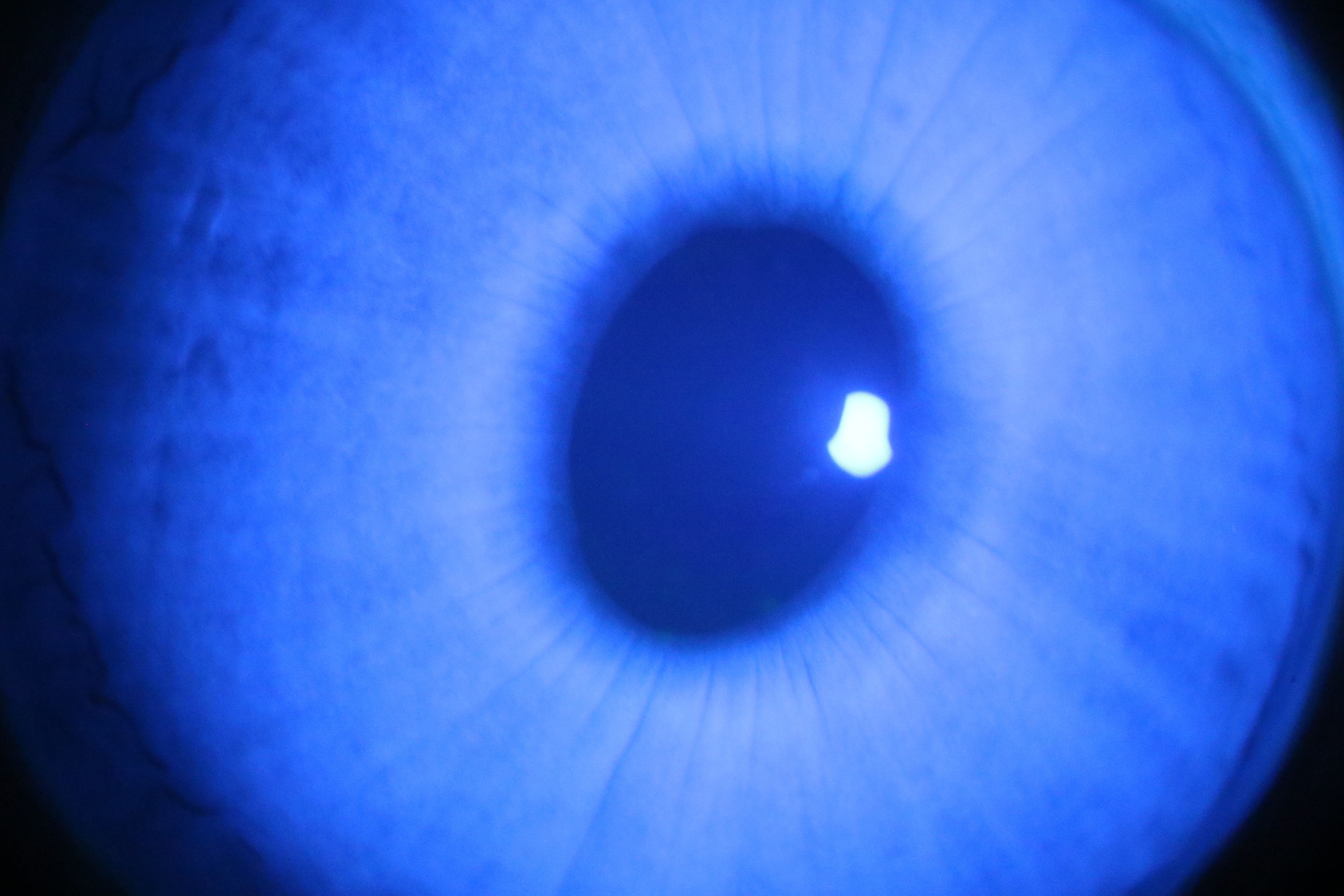

Supplement: Supplementary file 2 [file Data_Sheet_2.ZIP › corneal fluorescein staining/IMG_0251∩╝êNC∩╝ë.JPG]

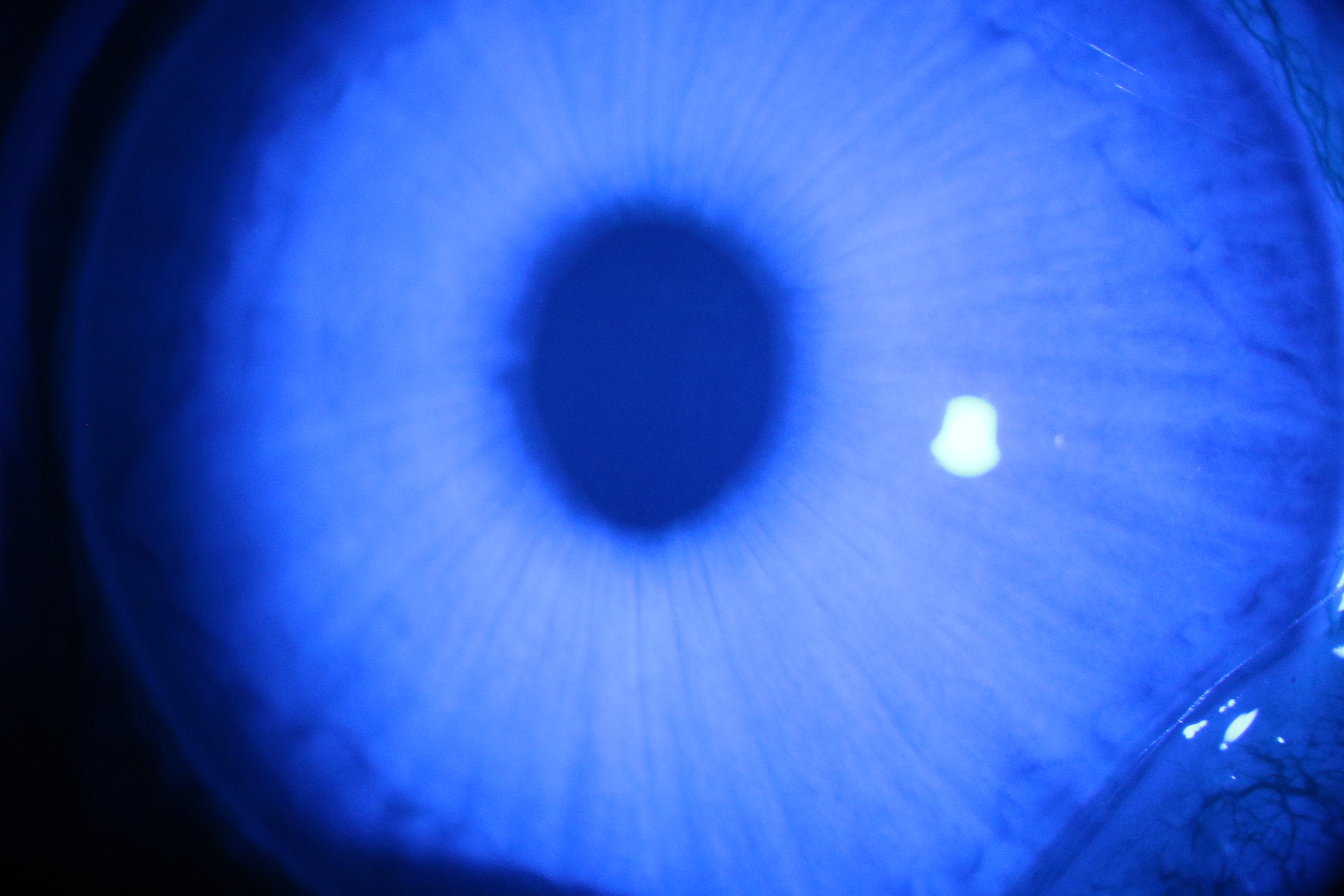

Supplement: Supplementary file 2 [file Data_Sheet_2.ZIP › corneal fluorescein staining/IMG_0418∩╝êTCXL+AC∩╝ë.JPG]

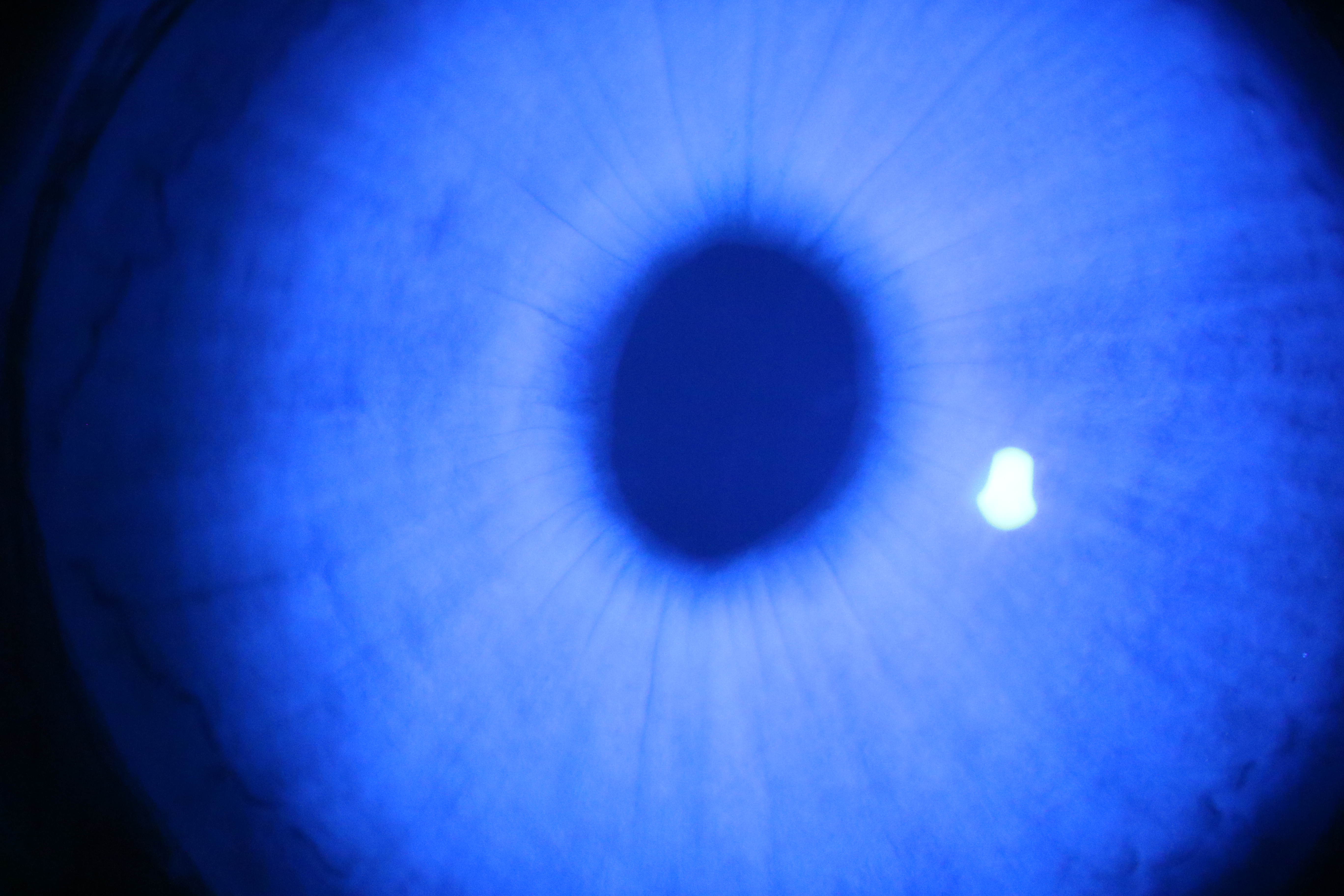

Supplement: Supplementary file 2 [file Data_Sheet_2.ZIP › corneal fluorescein staining/IMG_0299∩╝êTCXL+AC+Medication).JPG]

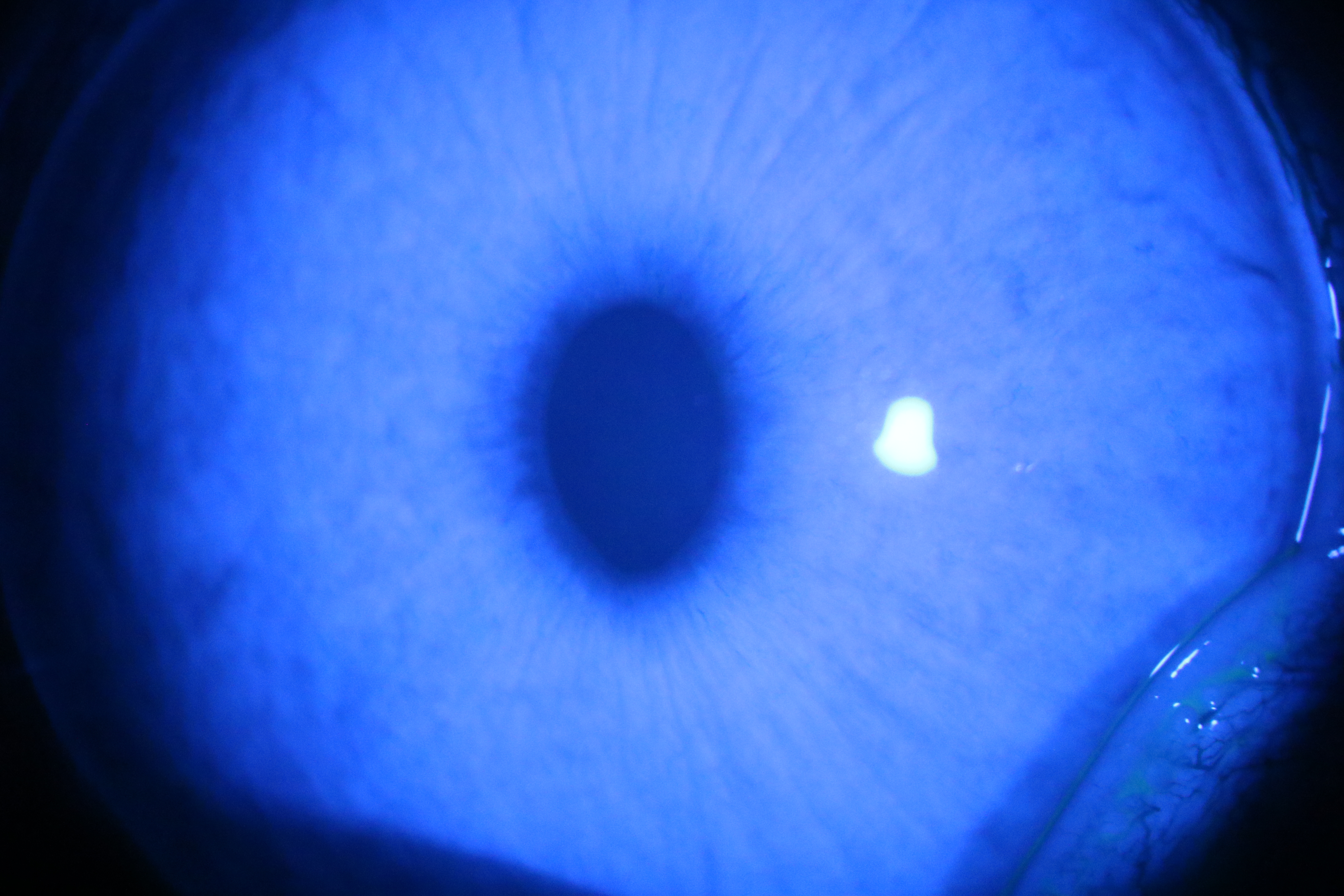

Supplement: Supplementary file 2 [file Data_Sheet_2.ZIP › corneal fluorescein staining/IMG_0185∩╝êTCXL∩╝ë.JPG]

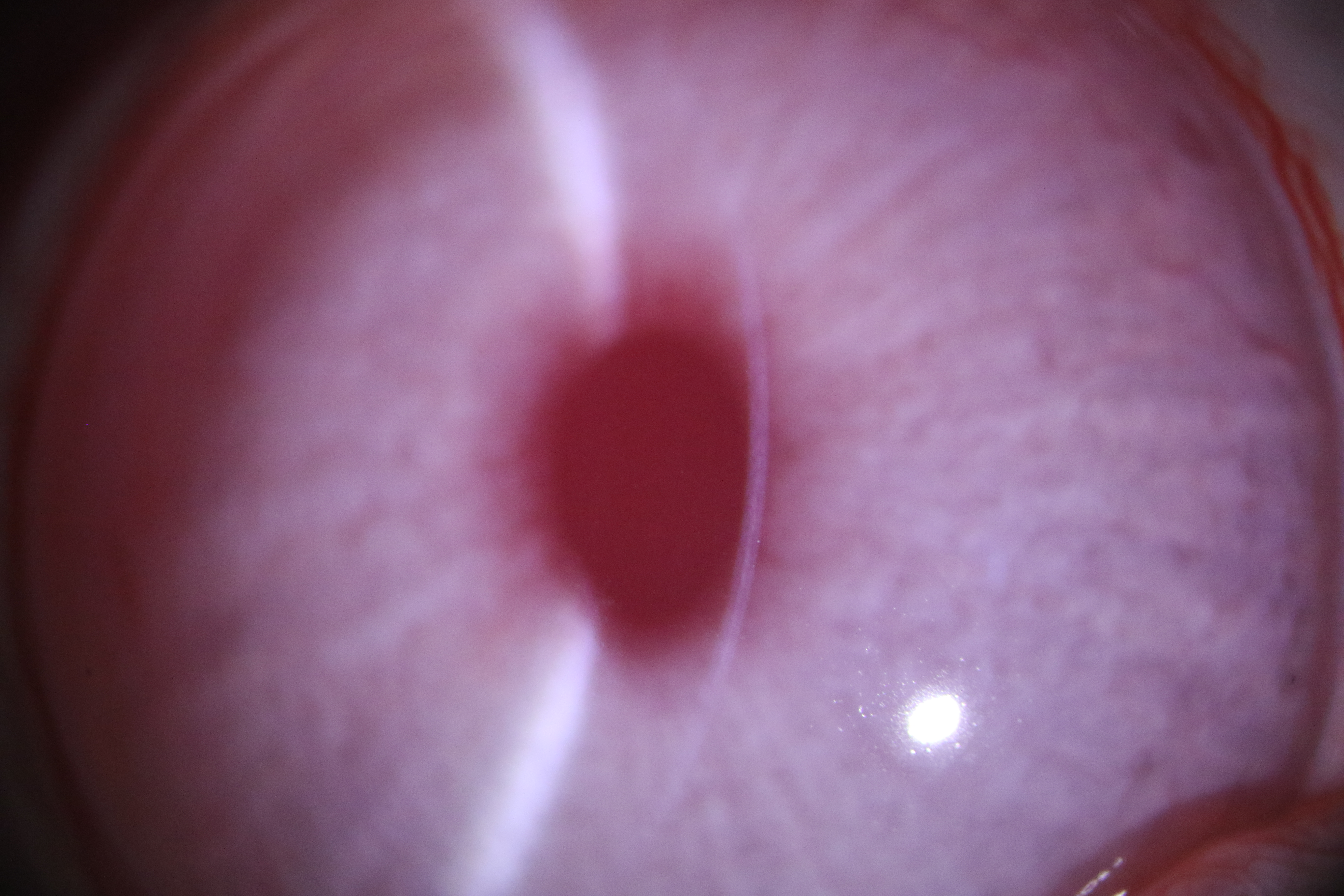

Supplement: Supplementary file 3 [file Data_Sheet_3.ZIP › demarcation line under slit lamp/TCXL.JPG]

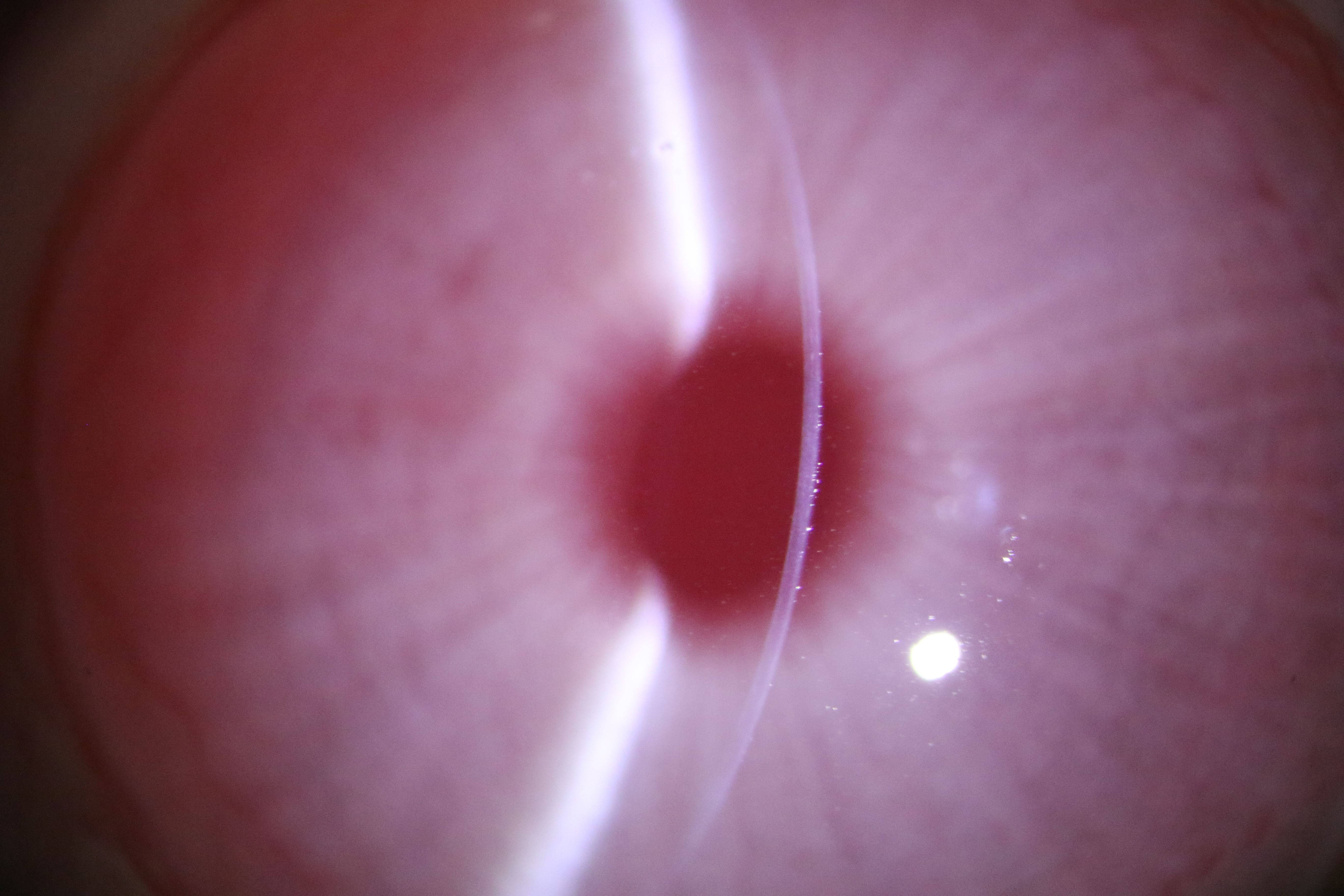

Supplement: Supplementary file 3 [file Data_Sheet_3.ZIP › demarcation line under slit lamp/TCXL+AC+PA.JPG]

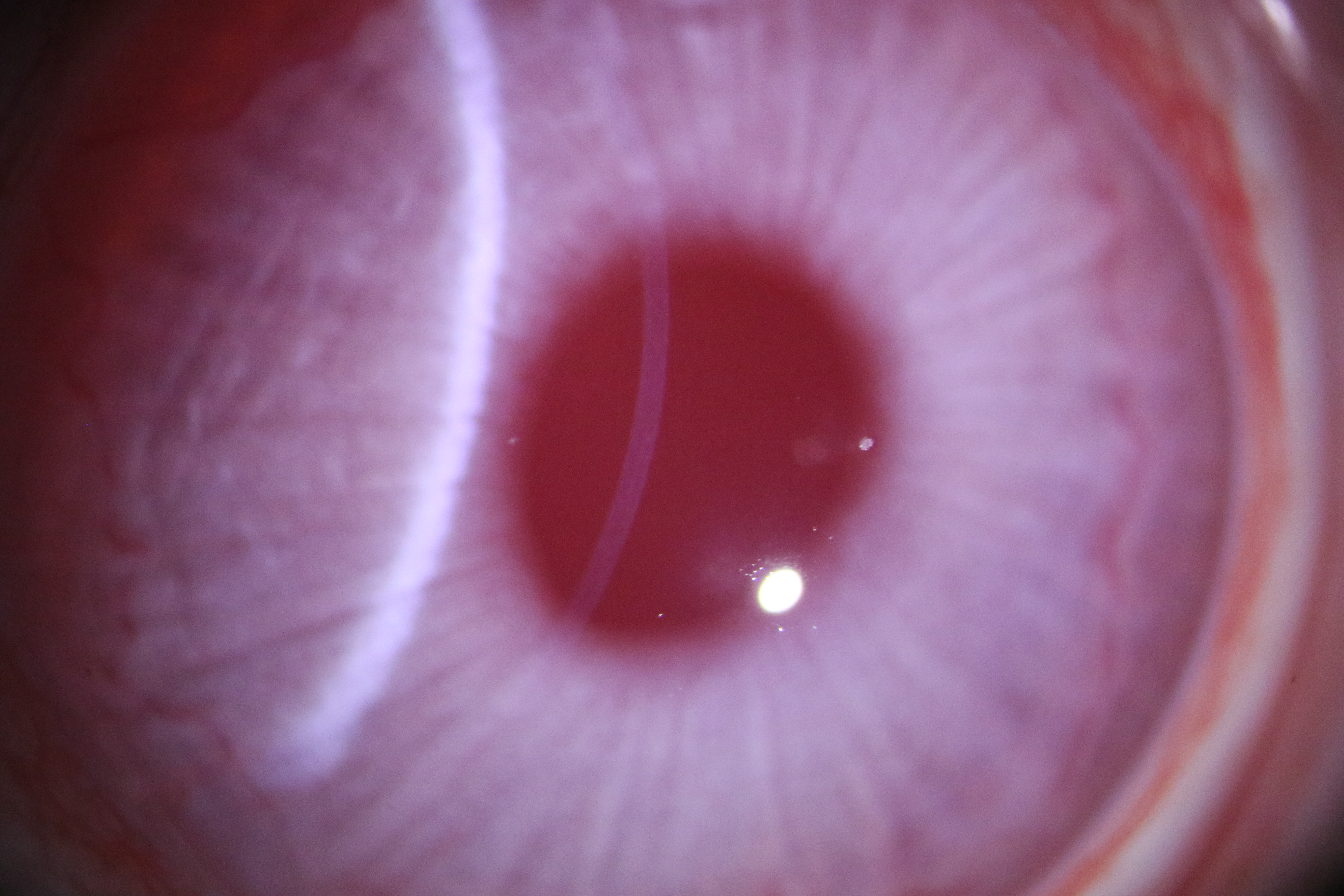

Supplement: Supplementary file 3 [file Data_Sheet_3.ZIP › demarcation line under slit lamp/NC(normal).JPG]

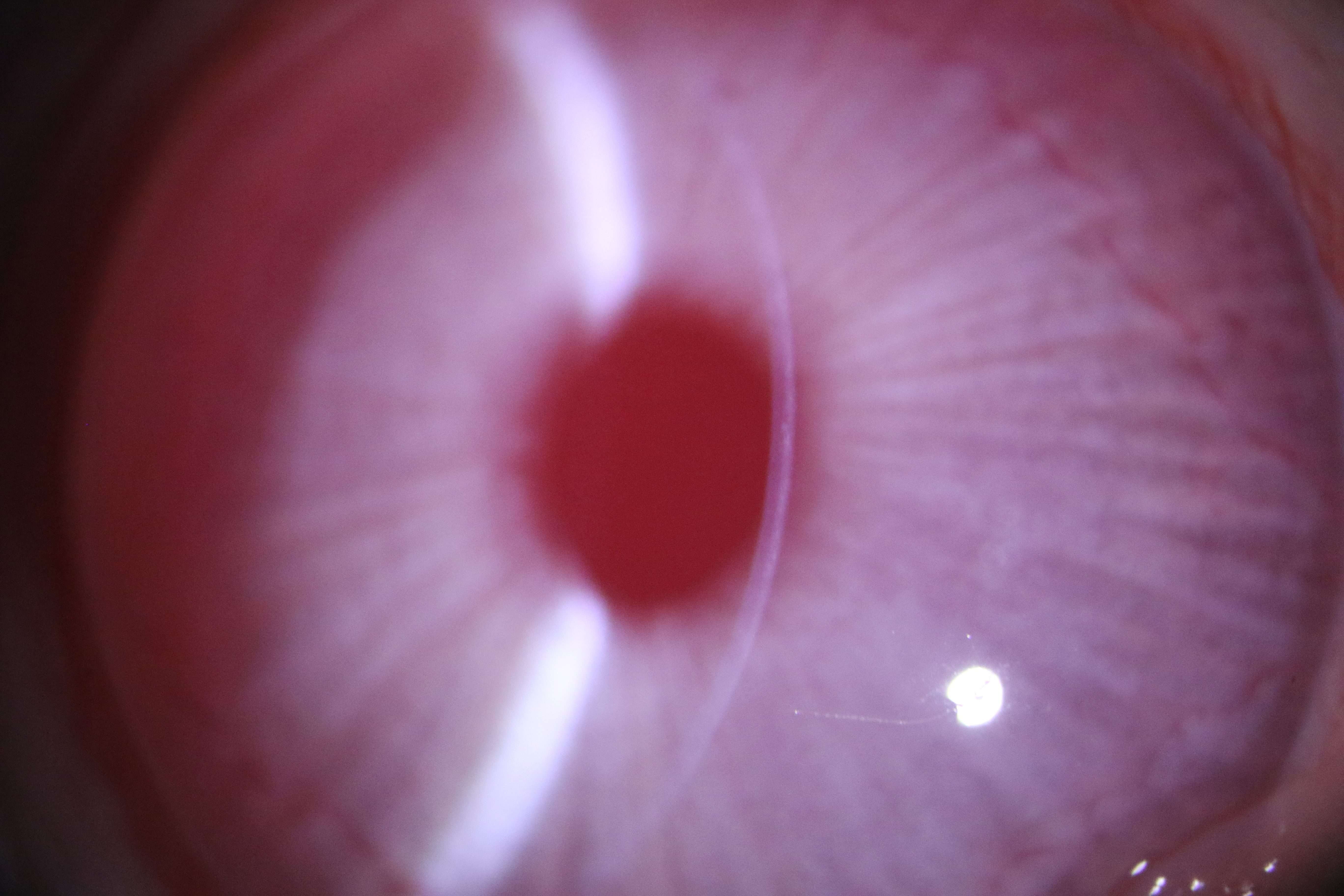

Supplement: Supplementary file 3 [file Data_Sheet_3.ZIP › demarcation line under slit lamp/TCXL+AC.JPG]

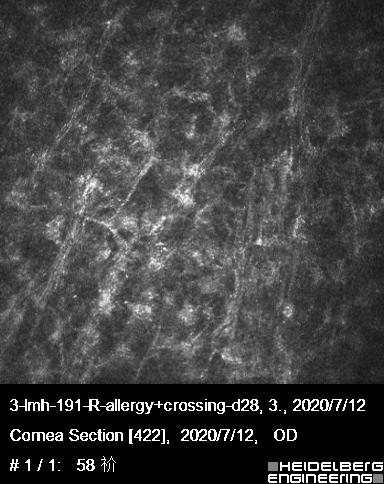

Supplement: Supplementary file 5 [file Data_Sheet_5.ZIP › 3-original images of HRT3/TCXL+AC∩╝ê191∩╝ë/3-lmh-191-R-allergy+crossing-d283(422).tif]

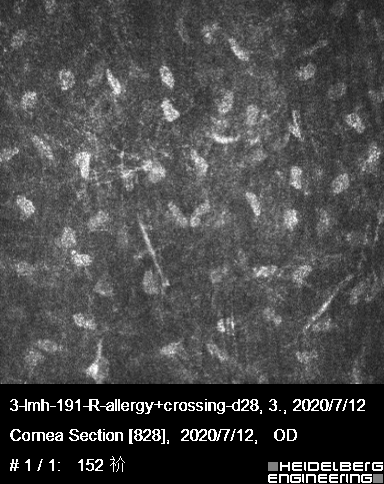

Supplement: Supplementary file 5 [file Data_Sheet_5.ZIP › 3-original images of HRT3/TCXL+AC∩╝ê191∩╝ë/3-lmh-191-R-allergy+crossing-d283(828).tif]

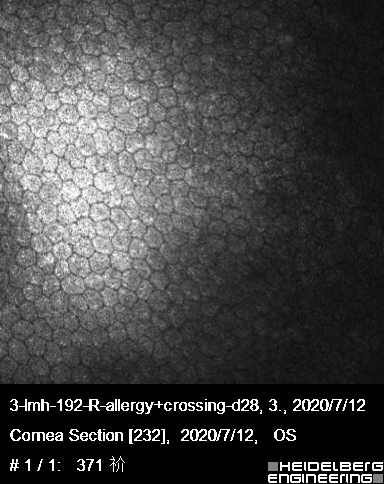

Supplement: Supplementary file 5 [file Data_Sheet_5.ZIP › 3-original images of HRT3/TCXL+AC∩╝ê191∩╝ë/3-lmh-192-R-allergy+crossing-d283(232).tif]

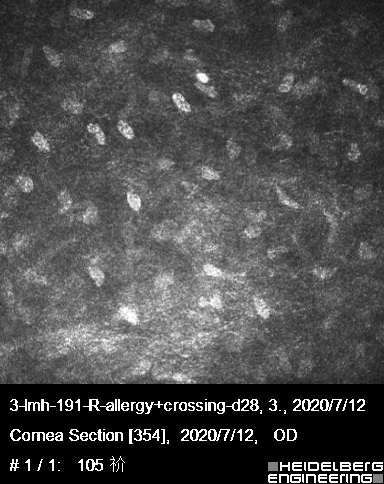

Supplement: Supplementary file 5 [file Data_Sheet_5.ZIP › 3-original images of HRT3/TCXL+AC∩╝ê191∩╝ë/3-lmh-191-R-allergy+crossing-d283(354).tif]

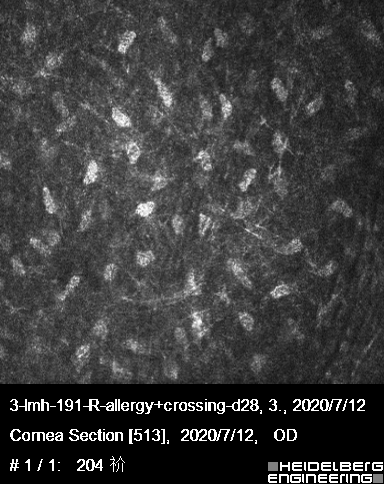

Supplement: Supplementary file 5 [file Data_Sheet_5.ZIP › 3-original images of HRT3/TCXL+AC∩╝ê191∩╝ë/3-lmh-191-R-allergy+crossing-d283(513).tif]

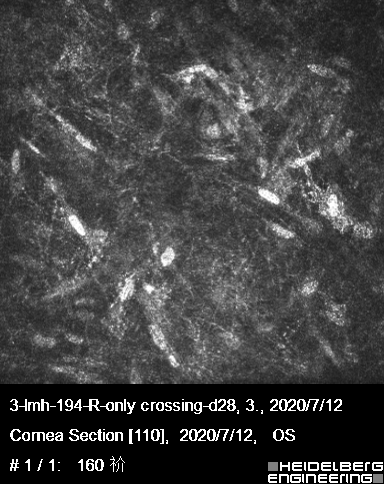

Supplement: Supplementary file 5 [file Data_Sheet_5.ZIP › 3-original images of HRT3/TCXL ∩╝ê194∩╝ë/3-lmh-194-R-only crossing-d283(110).tif]

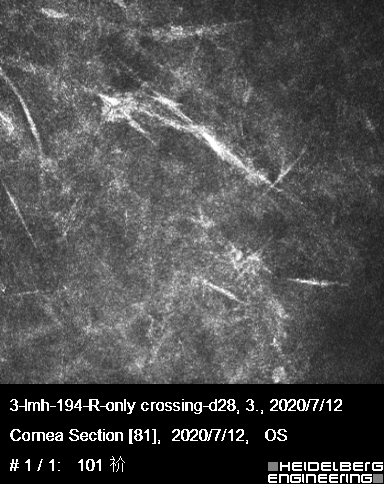

Supplement: Supplementary file 5 [file Data_Sheet_5.ZIP › 3-original images of HRT3/TCXL ∩╝ê194∩╝ë/3-lmh-194-R-only crossing-d283(81).tif]

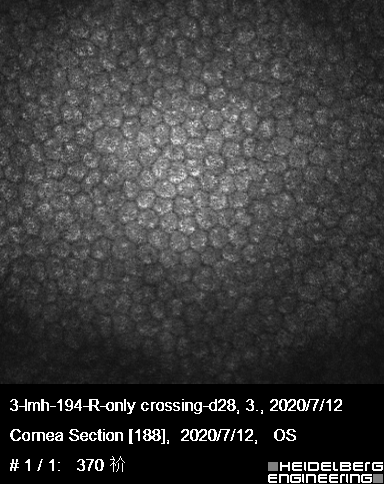

Supplement: Supplementary file 5 [file Data_Sheet_5.ZIP › 3-original images of HRT3/TCXL ∩╝ê194∩╝ë/3-lmh-194-R-only crossing-d283(188).tif]

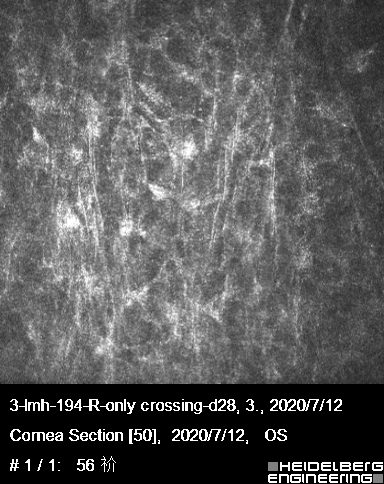

Supplement: Supplementary file 5 [file Data_Sheet_5.ZIP › 3-original images of HRT3/TCXL ∩╝ê194∩╝ë/3-lmh-194-R-only crossing-d283(50).tif]

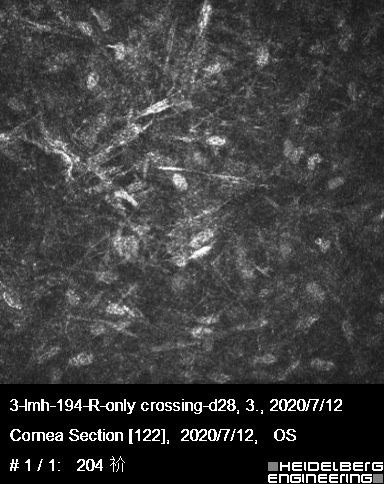

Supplement: Supplementary file 5 [file Data_Sheet_5.ZIP › 3-original images of HRT3/TCXL ∩╝ê194∩╝ë/3-lmh-194-R-only crossing-d283(122).tif]

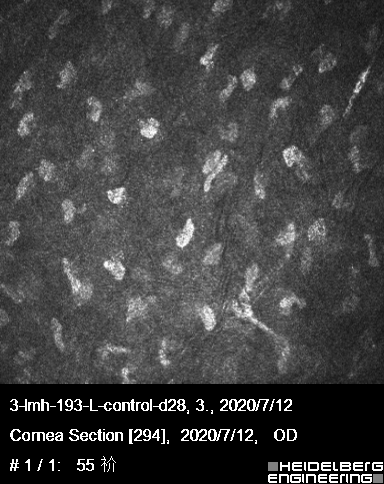

Supplement: Supplementary file 5 [file Data_Sheet_5.ZIP › 3-original images of HRT3/control/50-60-3-lmh-193-L-control-d283(294).tif]

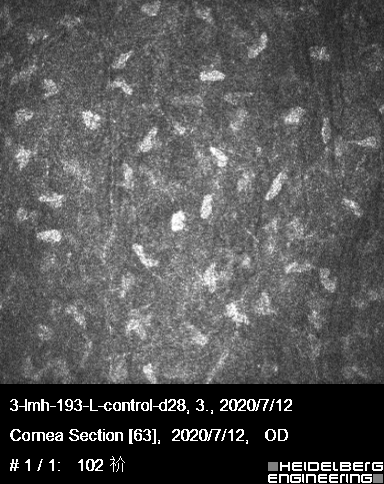

Supplement: Supplementary file 5 [file Data_Sheet_5.ZIP › 3-original images of HRT3/control/100-110-3-lmh-193-L-control-d283(63).tif]

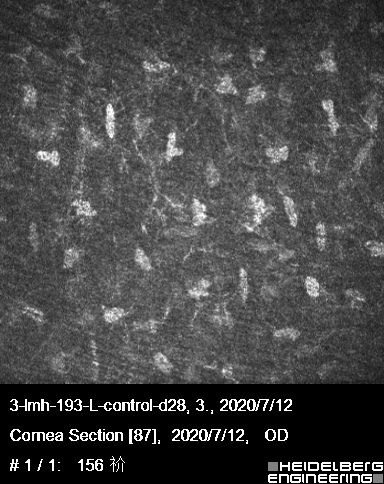

Supplement: Supplementary file 5 [file Data_Sheet_5.ZIP › 3-original images of HRT3/control/150-160-3-lmh-193-L-control-d283(87).tif]

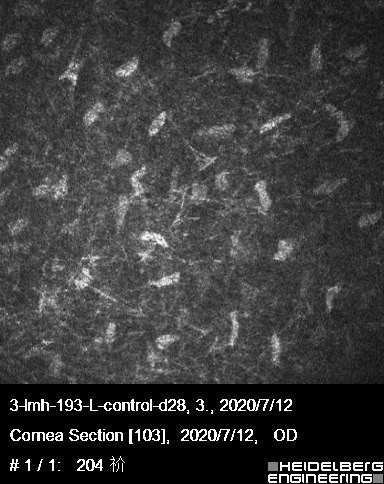

Supplement: Supplementary file 5 [file Data_Sheet_5.ZIP › 3-original images of HRT3/control/200-210-3-lmh-193-L-control-d283(103).tif]

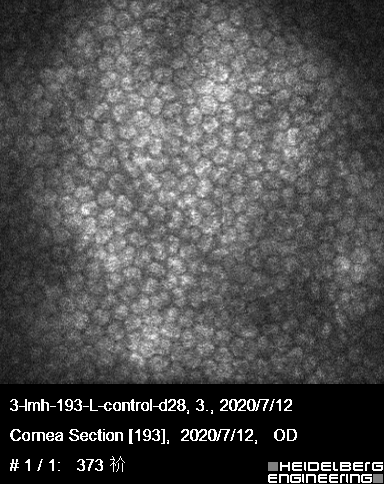

Supplement: Supplementary file 5 [file Data_Sheet_5.ZIP › 3-original images of HRT3/control/EC -350-3-lmh-193-L-control-d283(193).tif]

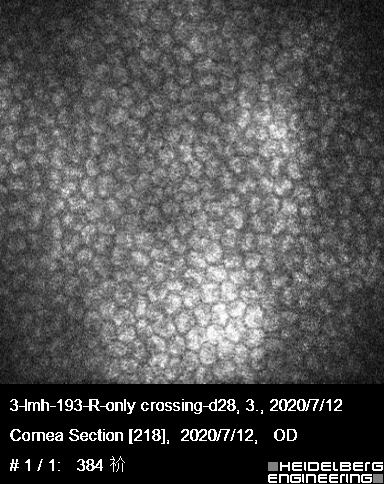

Supplement: Supplementary file 5 [file Data_Sheet_5.ZIP › 3-original images of HRT3/TCXL+AC+PA∩╝êTCXL 193∩╝ë/3-lmh-193-R-only crossing-d283(218).tif]

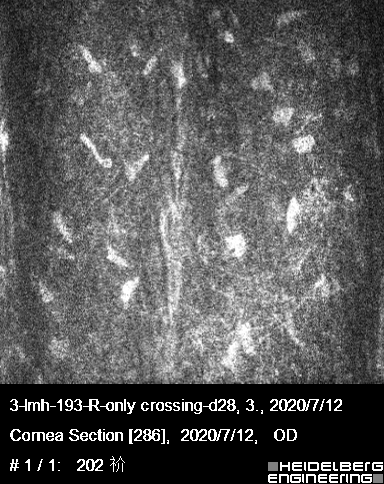

Supplement: Supplementary file 5 [file Data_Sheet_5.ZIP › 3-original images of HRT3/TCXL+AC+PA∩╝êTCXL 193∩╝ë/3-lmh-193-R-only crossing-d283(286).tif]

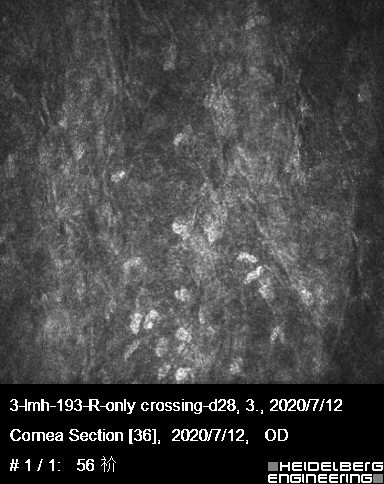

Supplement: Supplementary file 5 [file Data_Sheet_5.ZIP › 3-original images of HRT3/TCXL+AC+PA∩╝êTCXL 193∩╝ë/3-lmh-193-R-only crossing-d283(36).tif]

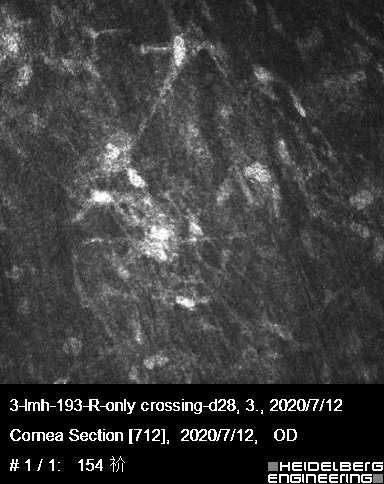

Supplement: Supplementary file 5 [file Data_Sheet_5.ZIP › 3-original images of HRT3/TCXL+AC+PA∩╝êTCXL 193∩╝ë/3-lmh-193-R-only crossing-d283(712).tif]

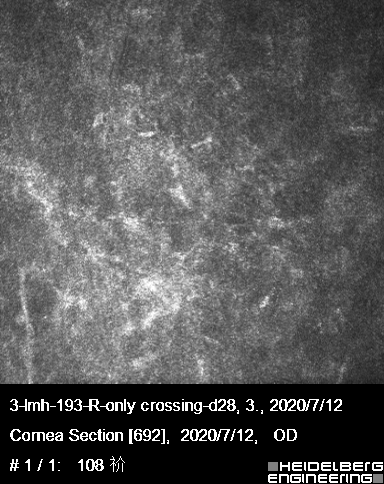

Supplement: Supplementary file 5 [file Data_Sheet_5.ZIP › 3-original images of HRT3/TCXL+AC+PA∩╝êTCXL 193∩╝ë/3-lmh-193-R-only crossing-d283(692).tif]

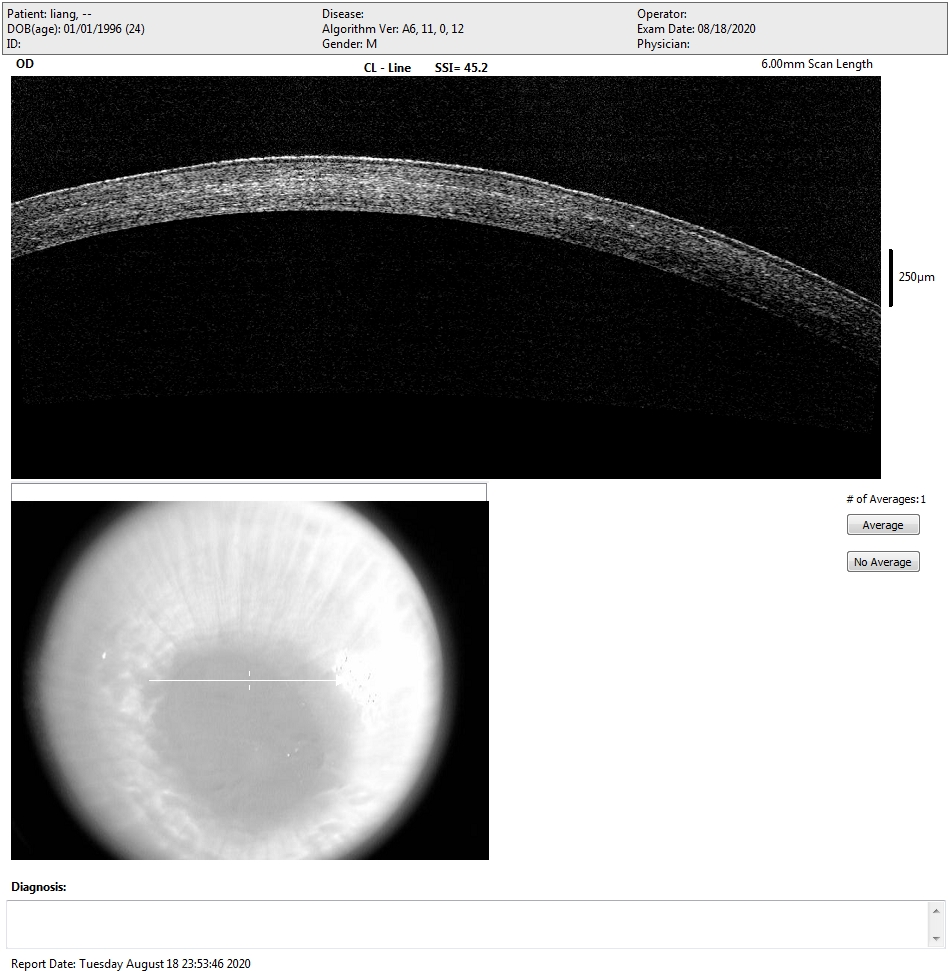

Supplement: Supplementary file 6 [file Data_Sheet_6.ZIP › 4-original images of AS-OCT/367od4 (TCXL).jpg]

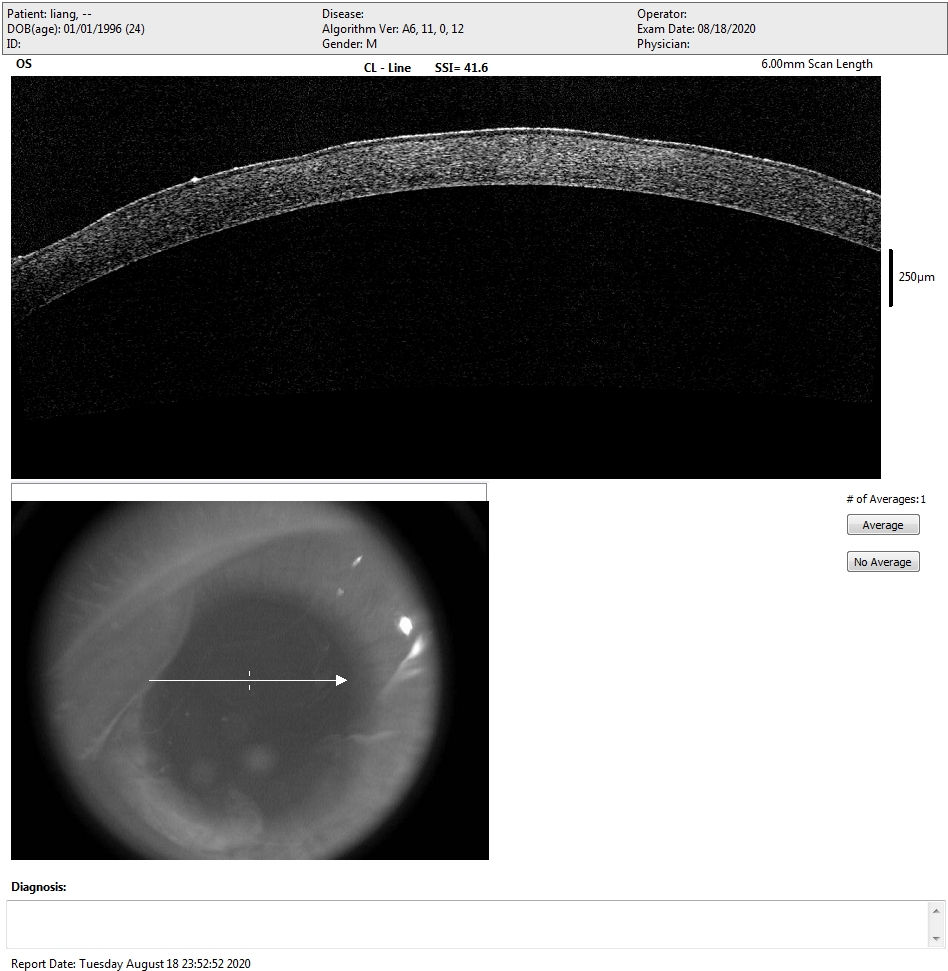

Supplement: Supplementary file 6 [file Data_Sheet_6.ZIP › 4-original images of AS-OCT/367os3∩╝ênormal control).jpg]

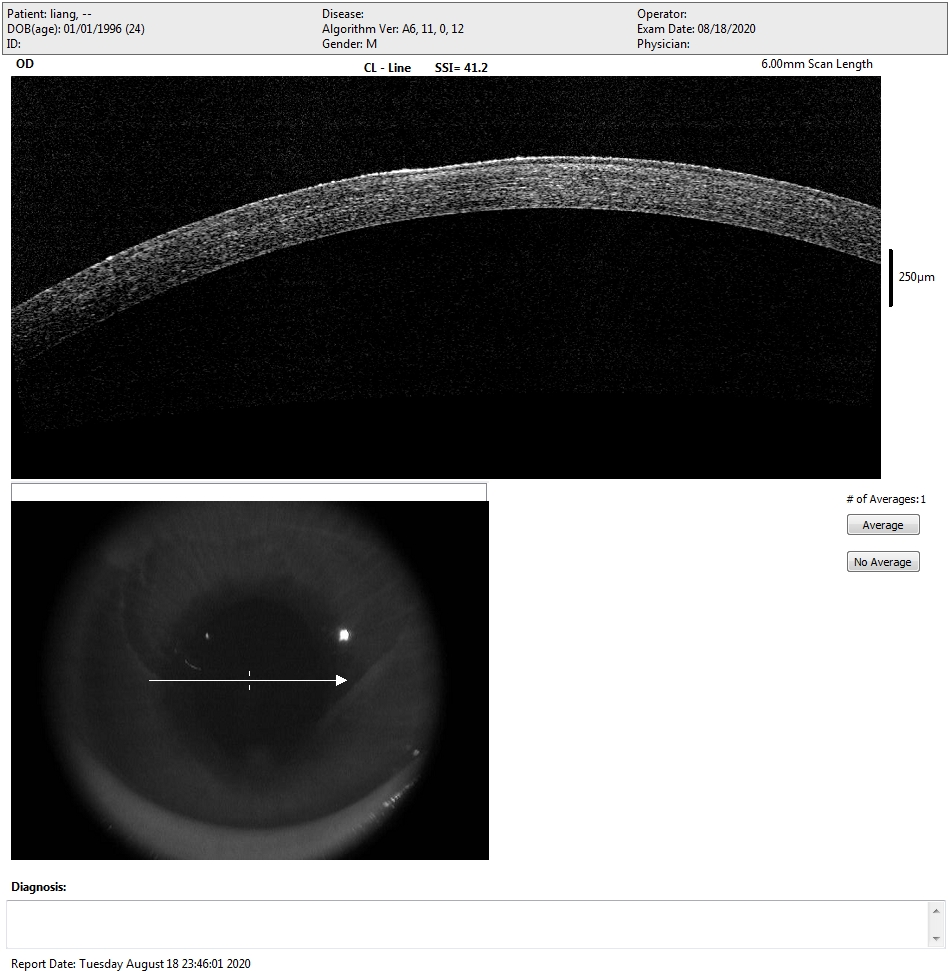

Supplement: Supplementary file 6 [file Data_Sheet_6.ZIP › 4-original images of AS-OCT/362od1 (TCXL+AC).jpg]

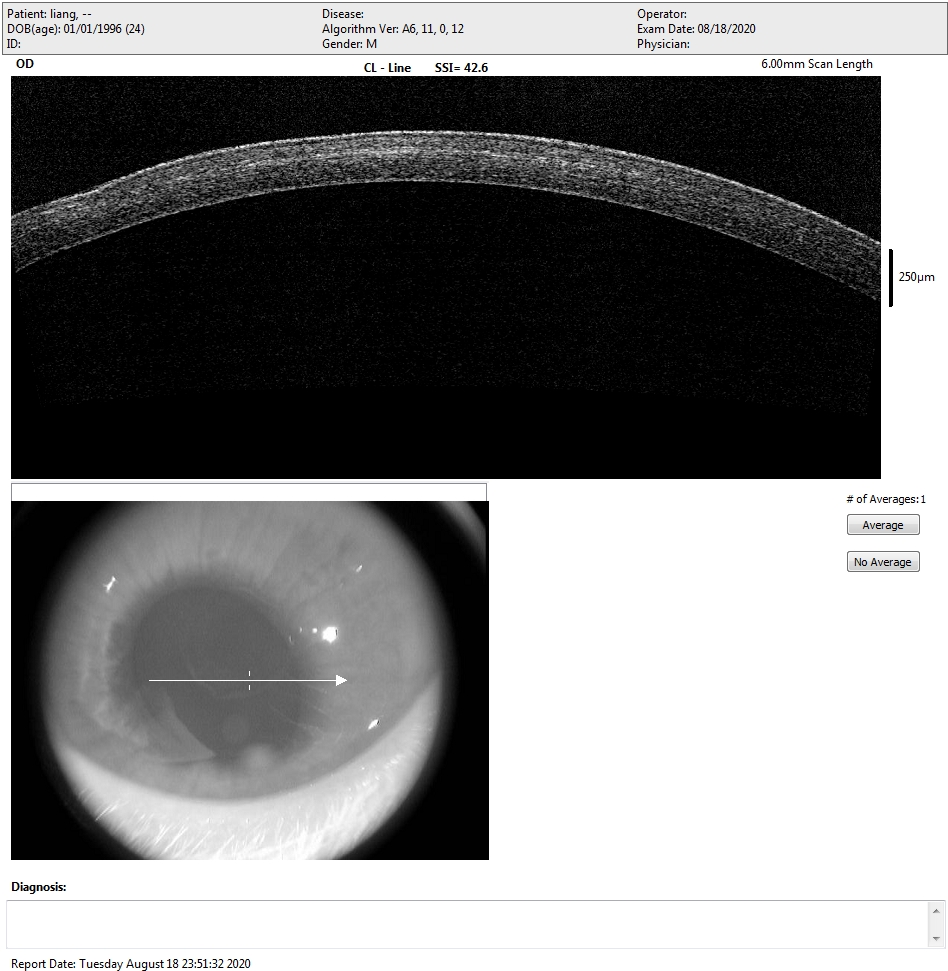

Supplement: Supplementary file 6 [file Data_Sheet_6.ZIP › 4-original images of AS-OCT/374od8∩╝êTCXL+AC+PA).jpg]

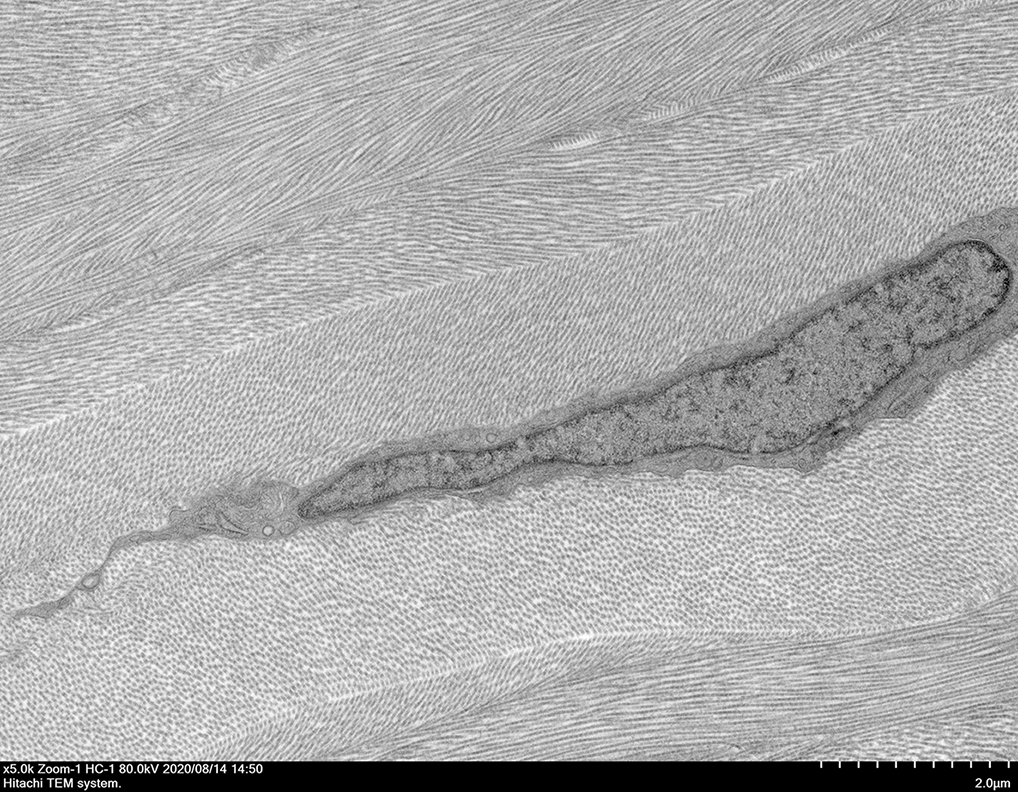

Supplement: Supplementary file 8 [file Image_1.TIF]

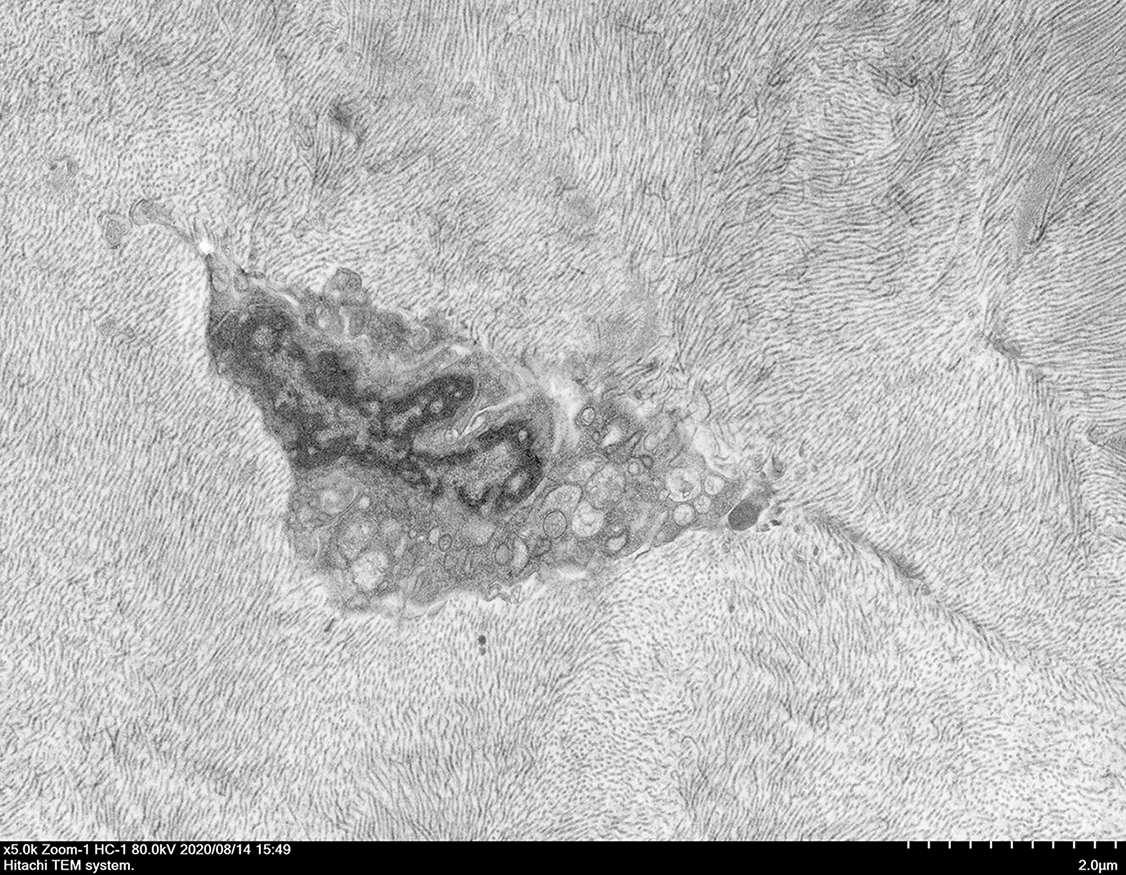

Supplement: Supplementary file 9 [file Image_2.TIF]

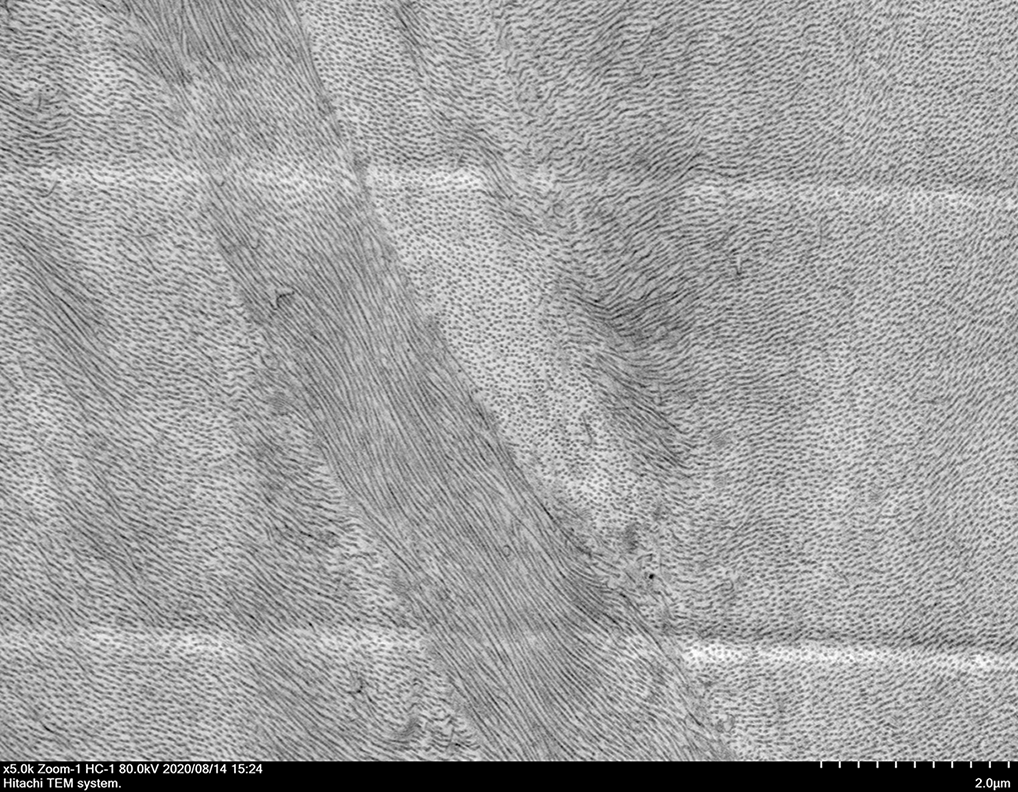

Supplement: Supplementary file 10 [file Image_3.TIF]

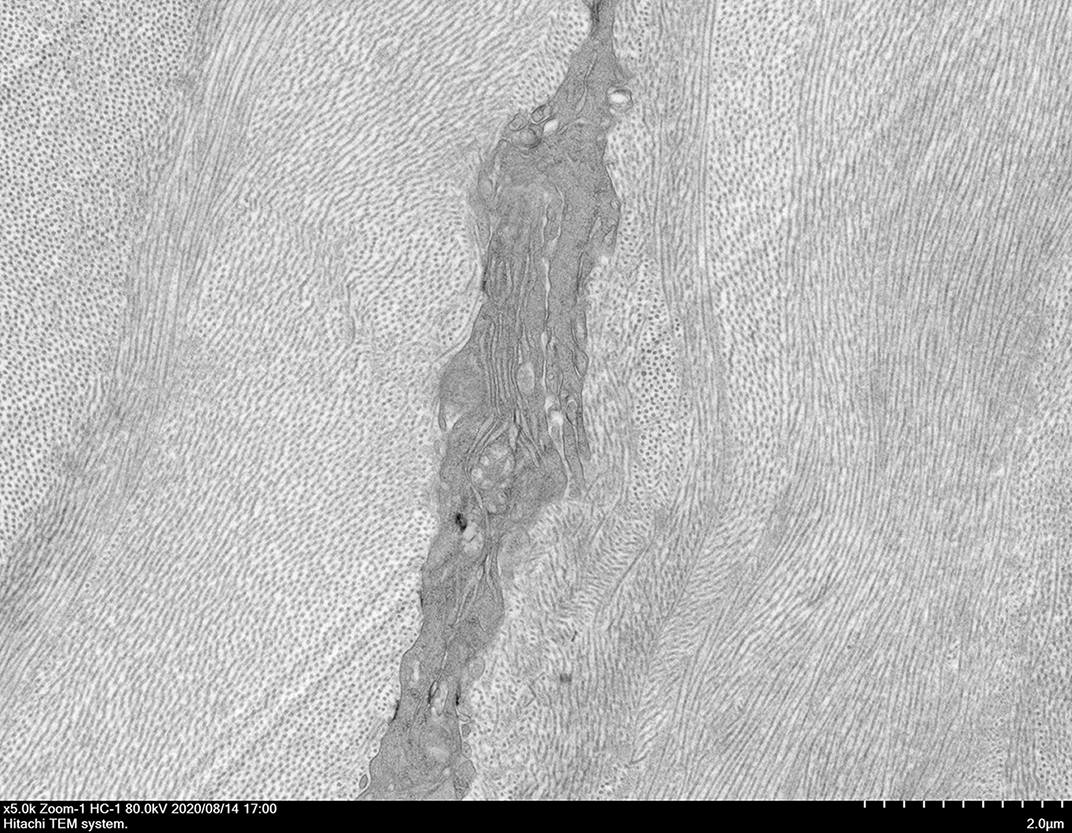

Supplement: Supplementary file 11 [file Image_4.TIF]

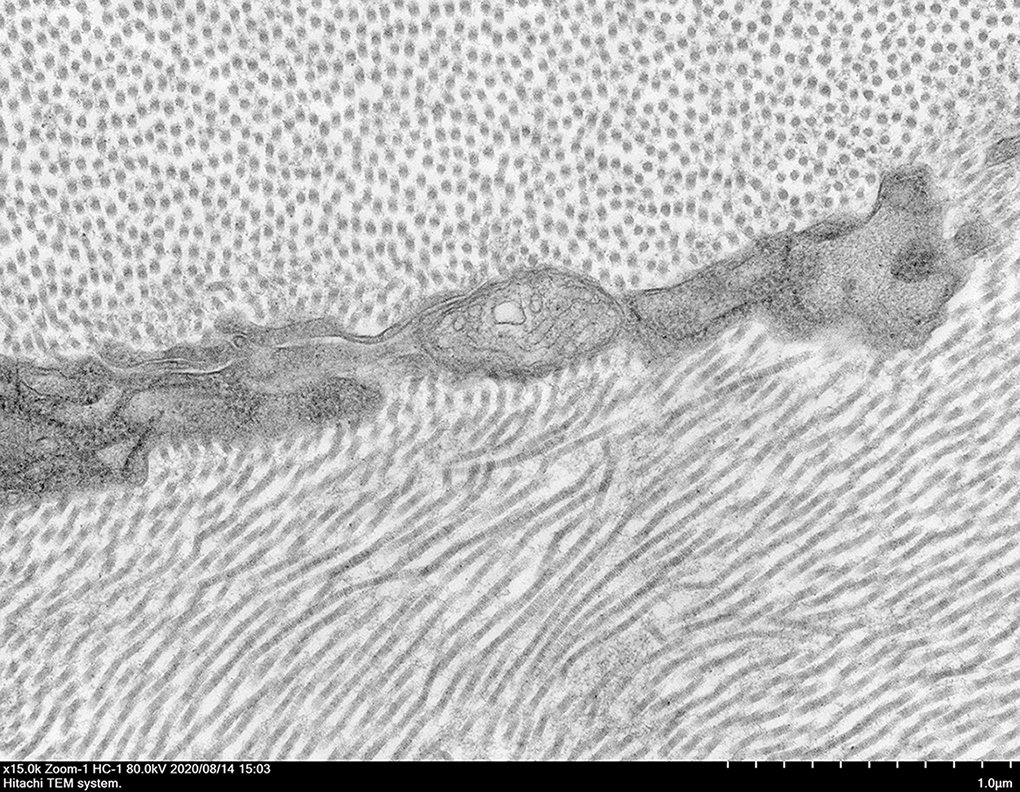

Supplement: Supplementary file 12 [file Image_5.TIF]

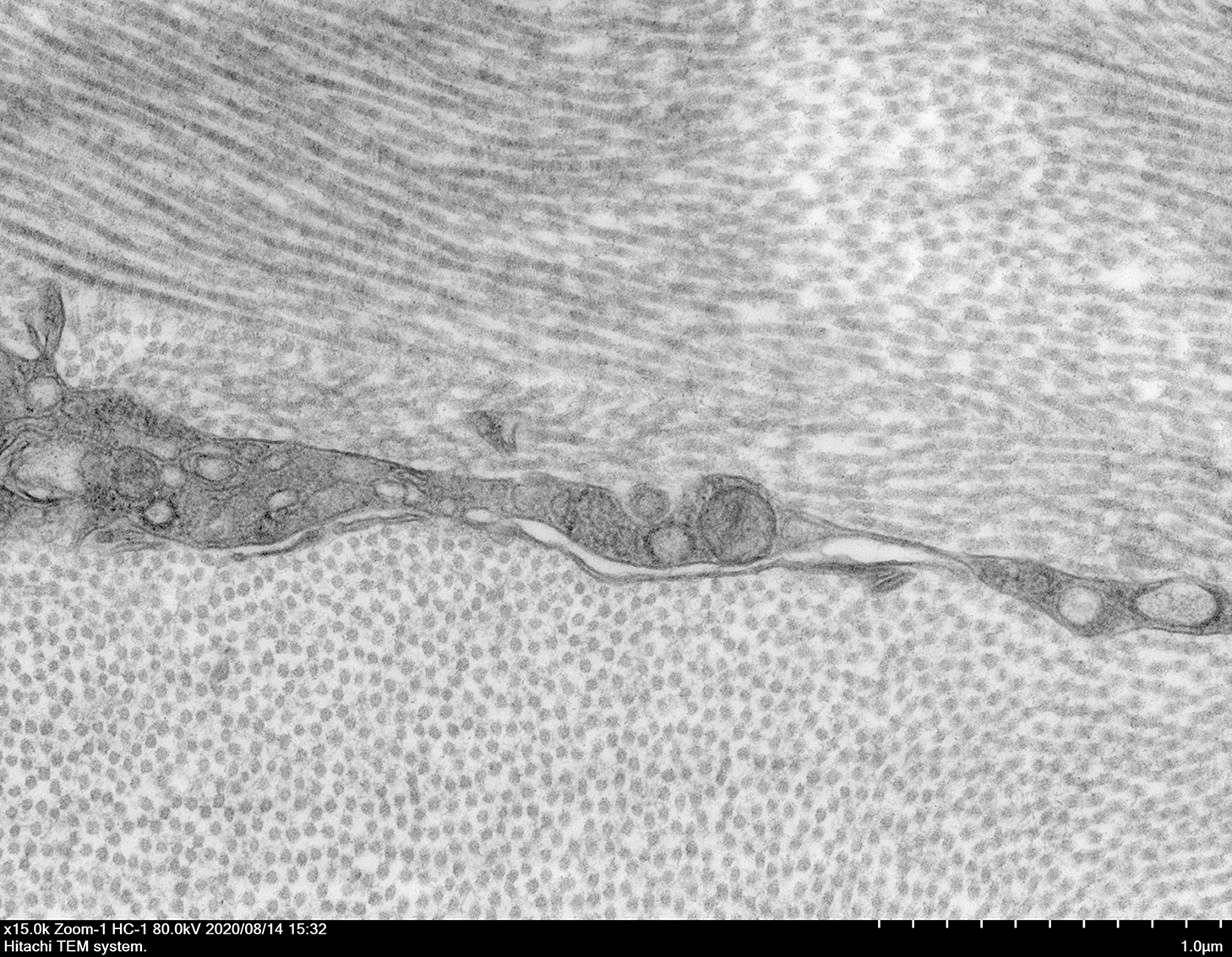

Supplement: Supplementary file 13 [file Image_6.TIF]

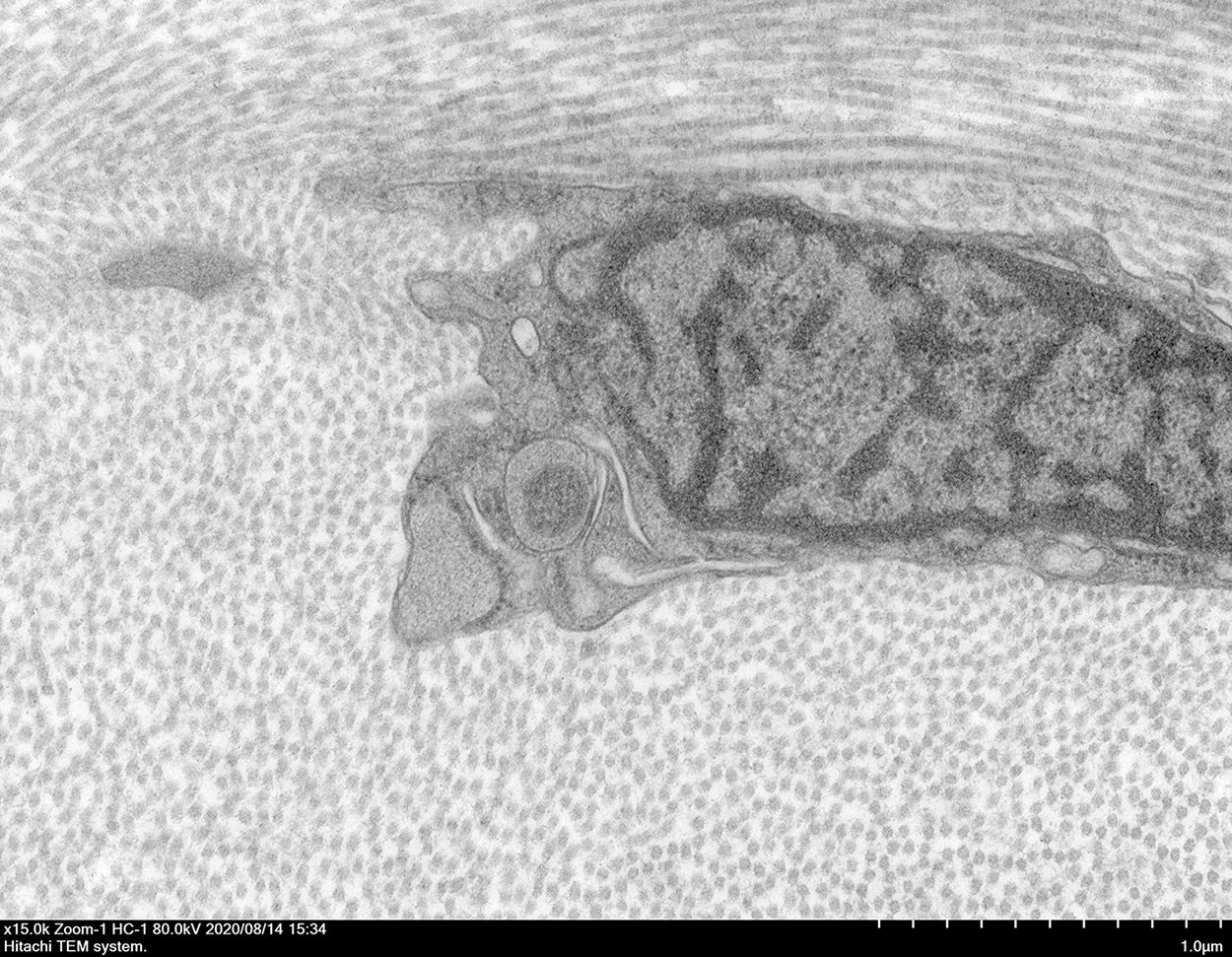

Supplement: Supplementary file 14 [file Image_7.TIF]

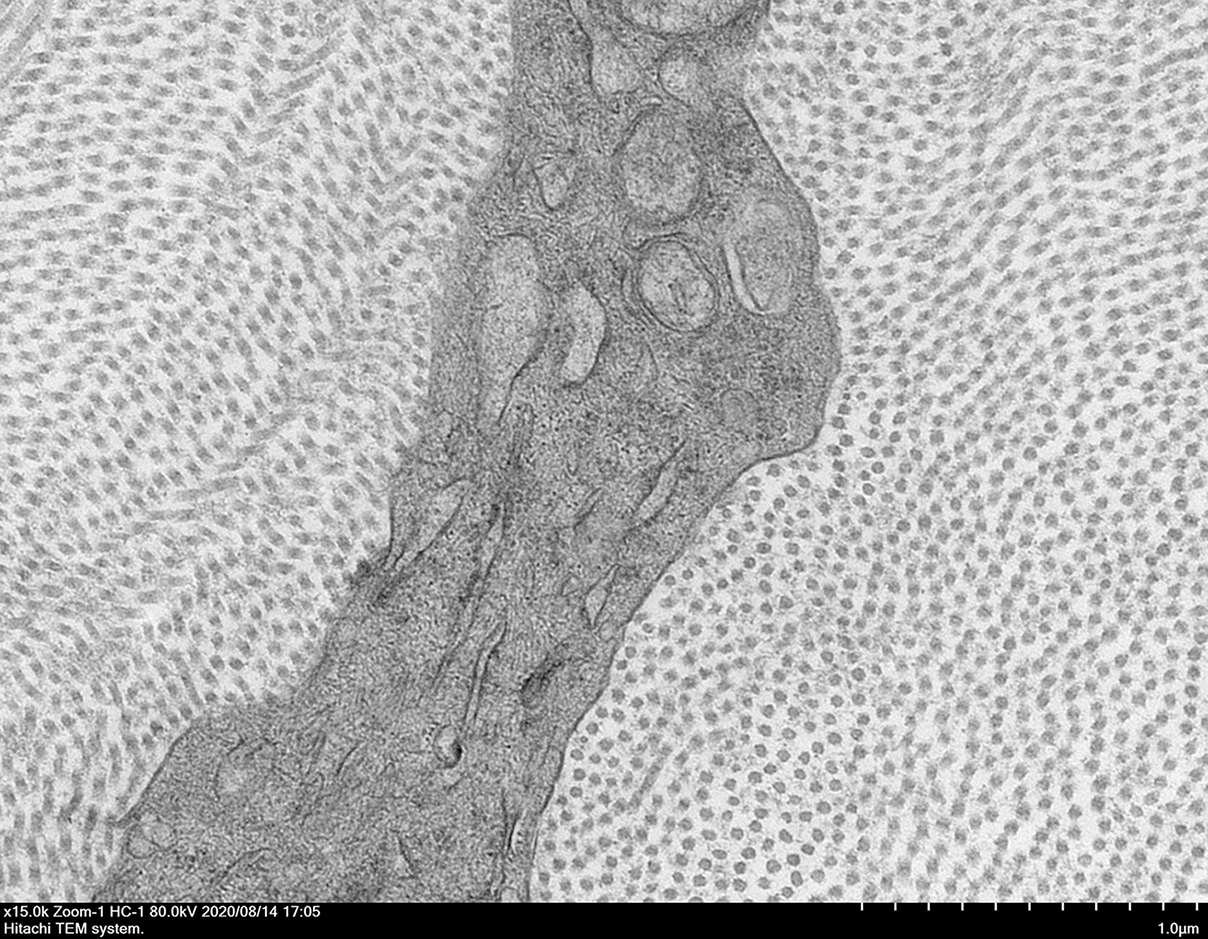

Supplement: Supplementary file 15 [file Image_8.TIF]
